# Anti-cancer therapy is associated with long-term epigenomic changes in childhood cancer survivors

**DOI:** 10.1038/s41416-022-01792-9

**Published:** 2022-03-30

**Authors:** Natassia Robinson, John Casement, Marc J. Gunter, Inge Huybrechts, Antonio Agudo, Miguel Rodríguez Barranco, Fabian Eichelmann, Theron Johnson, Rudolf Kaaks, Valeria Pala, Salvatore Panico, Torkjel M. Sandanger, Matthias B. Schultze, Ruth C. Travis, Rosario Tumino, Paolo Vineis, Elisabete Weiderpass, Roderick Skinner, Linda Sharp, Jill A McKay, Gordon Strathdee

**Affiliations:** 1 Newcastle University Centre for Cancer, Biosciences Institute, Newcastle University, Newcastle upon Tyne, UK; 2 Bioinformatic Support Unit, Newcastle University, Newcastle upon Tyne, UK; 3 Section of Nutrition and Metabolism, IARC, Lyon, France; 4 Unit of Nutrition and Cancer, Catalan Institute of Oncology - ICO, Nutrition and Cancer Group, Bellvitge Biomedical Research Institute - IDIBELL, L’Hospitalet de Llobregat, Barcelona, 08908 Spain; 5 Andalusian School of Public Health, ibs.GRANADA, CIBERESP, Granada, Spain; 6 German Institute of Human Nutrition Potsdam-Rehbrücke, Heidelberg, Germany; 7 German Cancer Research Center (DKFZ), Heidelberg, Germany; 8 Fondazione IRCCS Istituto Nazionale dei Tumori di Milano, Milan, Italy; 9 Dipartimento di Medicina Clinica e Chirurgia, Federico II University, Naples, Italy; 10 Health Faculty, UiT-the Arctic University of Norway, Tromsø, Norway; 11 Cancer Epidemiology Unit, University of Oxford, Oxford, UK; 12 Cancer Registry and Histopathology Department Azienda Sanitaria Provinciale (ASP), Ragusa, Italy; 13 MRC Centre for Environment and Health, Imperial College London, London, UK; 14 International Agency for Research on Cancer, Lyon, France; 15 Newcastle University Centre for Cancer, Translational and Clinical Research Institute, Newcastle University, Newcastle upon Tyne, UK; 16 Department of Paediatric and Adolescent Haematology/Oncology, Great North Children’s Hospital, Newcastle, UK; 17 Newcastle University Centre for Cancer, Population Health Sciences Institute, Newcastle upon Tyne, UK; 18 Department of Applied Sciences, Northumbria University, Newcastle upon Tyne, UK

**Keywords:** Paediatric cancer, Cancer therapy, DNA methylation

## Abstract

**Background:**

Childhood cancer survivors (CCS) exhibit significantly increased chronic diseases and premature death. Abnormalities in DNA methylation are associated with development of chronic diseases and reduced life expectancy. We investigated the hypothesis that anti-cancer treatments are associated with long-term DNA methylation changes that could be key drivers of adverse late health effects.

**Methods:**

Genome-wide DNA methylation was assessed using MethylationEPIC arrays in paired samples (before/after therapy) from 32 childhood cancer patients. Separately, methylation was determined in 32 samples from different adult CCS (mean 22-years post-diagnosis) and compared with cancer-free controls (*n* = 284).

**Results:**

Widespread DNA methylation changes were identified post-treatment in childhood cancer patients, including 146 differentially methylated regions (DMRs), which were consistently altered in the 32 post-treatment samples. Analysis of adult CCS identified matching methylation changes at 107/146 of the DMRs, suggesting potential long-term retention of post-therapy changes. Adult survivors also exhibited epigenetic age acceleration, independent of DMR methylation. Furthermore, altered methylation at the *DUSP6* DMR was significantly associated with early mortality, suggesting altered methylation may be prognostic for some late adverse health effects in CCS.

**Conclusions:**

These novel methylation changes could serve as biomarkers for assessing normal cell toxicity in ongoing treatments and predicting long-term health outcomes in CCS.

## Background

Treatment of childhood cancer has improved dramatically in recent years and overall survival rates for childhood cancers exceed 80% in high-income countries [1]. Thus, the population of adult-aged childhood cancer survivors (CCS) continues to increase, representing approximately 1 in 750 young adults in the UK [2]. However, childhood cancer patients generally receive aggressive multi-agent therapy and extensive follow-up of CCS has revealed their increased risk of long-term adverse health outcomes [3]. CCS have higher rates of many chronic diseases including a 3.6 to 6.4-fold increased risk of second cancers [4], and a 3–4-fold increased risk of cardiac mortality [5, 6]. These, in turn, are associated with highly elevated levels of morbidity and mortality and substantially reduced life expectancy for CCS [4]. Molecular markers for predicting late effects would be of significant clinical utility, both in enabling more targeted healthcare follow-up for those at greatest risk of significant late effects, and also for monitoring the long-term health impacts of novel therapies or treatment protocols.

One potential mechanism that may contribute to long-term health effects in cancer survivors is epigenetic changes induced by cancer therapies. DNA methylation is a key epigenetic modification that has important roles in control of gene expression and suppression of repetitive elements [7]. Furthermore, changes in DNA methylation are crucial in cancer development [7]. In addition, specific patterns of altered DNA methylation have been associated with all commonly occurring human chronic diseases [8], and age associated changes in DNA methylation have been shown to predict the development of multiple chronic diseases and reduced life expectancy [9]. Mendelian randomisation studies have found evidence that some of these DNA methylation changes are causal in the development of chronic diseases [10]. Indeed, multiple studies in humans and animal models have demonstrated that exposures in early life (e.g., cigarette smoke [11], starvation conditions [12], and sexual/physical abuse [13, 14]) can lead to alterations in DNA methylation, which are maintained into adulthood and predict increased risk of chronic disease. Using a candidate gene approach, we previously determined that exposure to chemotherapy can lead to increased levels of site-specific DNA methylation at the *HOXA4* locus [15]. We hypothesised that exposure of childhood cancer patients to cancer therapies may lead to aberrant DNA methylation in normal cells, which could be maintained lifelong and thus contribute to increased levels of chronic diseases and reduced life expectancy in CCS.

To examine this hypothesis, we first identified genome-wide changes in DNA methylation in childhood cancer patients following anti-cancer treatments (study-1). Subsequently, we examined the presence of the same DNA methylation changes in a different group of adult survivors of childhood cancer many years post-treatment, and their capacity to predict survival in CCS (study-2). The findings thus have potential to advance understanding of mechanisms underlying late effects in CCS and inform strategies to prevent or reduce life threatening or life changing late effects.

## Methods

### Study 1

To identify genome wide changes in DNA methylation following exposure to anti-cancer treatment we examined DNA methylation in normal, non-cancer cells in paired samples from childhood cancer patients before and after therapy. Samples were originally collected to assess the impact of exposure to a topoisomerase II inhibitor, as part of multi-drug regimens, which varied in the specific drugs, doses and treatment schedules. Patients were recruited between 1998–2000. Treatment details for these patients have been previously described [16] and are summarised in Supplementary Table 1. Patients included 32 children (between 1 and 15 years) with haematological cancers (*n* = 25; acute lymphoblastic leukaemia (ALL, *n* = 22), acute myeloid leukaemia (AML, *n* = 2), lymphoma *n* = 1), or solid tumours (*n* = 7: 5 neuroblastoma, 1 osteosarcoma, 1 Wilms tumour) (Table 1). Sets of paired samples (either blood or bone marrow) were taken at two time points referred to as “before” and “after” therapy (Table 1). To ensure that the methylation pattern was derived from non-cancer cells, for haematological malignancy patients, the “before” therapy sample had to be taken after remission was achieved (5–20 weeks of post-diagnosis). However, the treatment period for these malignancies is between 6 month and 2–3 years and treatment is not ceased after remission is achieved. For solid tumours, “before” samples were from at/near diagnosis, as blood/bone marrow samples from these patients contain negligible tumour material. “After” therapy samples for all CCS were taken 9–95 weeks after the “before” sample (mean 43.4 weeks between sample pairs) (specific details for each set of paired samples in Table 1). DNA was extracted using standard procedures [15].

**Table 1:** Summary of the childhood patient sample (study 1, recruited between 1998 and 2000).

Sample ID	Sex	Cancer type	Solid or Haem	Tissue	Early sample timing^a^	Late sample timing^a^	Early DNA methylation age (years)	Late DNA methylation age (years)	Change DNA methylation age (years)	Average DMR methylation^b^
**1**	Male	ALL	Haem	Blood	22 weeks	104 weeks	3.85	5.44	1.59	0.038
**2**	Female	ALL	Haem	Blood	13 weeks	104 weeks	6.97	6.65	−0.32	0.023
**3**	Female	ALL	Haem	Blood	9 weeks	104 weeks	3.87	4.40	0.54	0.065
**4**	Male	ALL	Haem	Blood	13 weeks	104 weeks	4.94	4.20	−0.74	0.043
**5**	Male	ALL	Haem	Blood	13 weeks	35 weeks	2.14	2.89	0.75	−0.011
**6**	Female	ALL	Haem	Blood	13 weeks	65 weeks	3.43	3.29	−0.14	0.009
**7**	Female	ALL	Haem	Blood	22 weeks	104 weeks	0.88	1.95	1.08	0.028
**8**	Male	AML	Haem	Blood	5/6 weeks	14 weeks	2.76	2.27	−0.49	0.043
**9**	Female	ALL	Haem	Blood	13 weeks	104 weeks	2.01	3.46	1.46	0.024
**10**	Male	ALL	Haem	Blood	26 weeks	104 weeks	2.72	3.90	1.18	0.014
**11**	Female	ALL	Haem	BM^c^	8 weeks	23 weeks	3.72	2.27	−1.44	0.068
**12**	Male	ALL	Haem	BM	8 weeks	59 weeks	2.48	3.20	0.72	0.074
**13**	Female	ALL	Haem	Blood	8 weeks	59 weeks	7.32	4.78	−2.53	0.060
**14**	Male	ALL	Haem	Blood	8 weeks	59 weeks	5.91	5.58	−0.32	0.039
**15**	Male	ALL	Haem	Blood	6 weeks	59 weeks	2.52	3.83	1.32	0.036
**16**	Male	Lymphoma	Haem	Blood	5 weeks	26 weeks	9.47	6.27	−3.20	0.057
**17**	Male	ALL	Haem	Blood	6 weeks	63 weeks	15.39	12.86	−2.53	0.059
**18**	Male	ALL	Haem	Blood	6 weeks	20 weeks	5.74	3.09	−2.65	0.036
**19**	Male	ALL	Haem	Blood	8 weeks	52 weeks	4.02	4.30	0.28	0.021
**20**	Male	Solid	Solid	BM	0 weeks	35 weeks	2.58	2.61	0.03	0.101
**21**	Female	ALL	Haem	BM	5 weeks	23 weeks	30.86	12.01	−18.85	0.059
**22**	Male	ALL	Haem	Blood	23 weeks	63 weeks	6.23	4.83	−1.39	0.012
**23**	Male	ALL	Haem	BM	8 weeks	40 weeks	10.53	9.78	−0.76	0.061
**24**	Male	Solid	Solid	Blood	1 week	30 weeks	5.13	3.58	−1.54	0.054
**25**	Female	AML	Haem	BM	5 weeks	26 weeks	3.04	2.85	−0.19	0.101
**26**	Male	Solid	Solid	Blood	0.5 week	20 weeks	6.23	3.20	−3.02	0.104
**27**	Female	ALL	Haem	BM	5 weeks	51 weeks	4.04	2.91	−1.12	0.087
**28**	Female	Solid	Solid	BM	0 weeks	20 weeks	3.19	2.08	−1.11	0.067
**29**	Male	ALL	Haem	Blood	20 weeks	52 weeks	4.09	4.90	0.82	0.016
**30**	Female	Solid	Solid	Blood	3 weeks	15 weeks	3.30	2.57	−0.73	0.055
**31**	Male	Solid	Solid	Blood	11 weeks	32 weeks	3.72	4.38	0.67	0.016
**32**	Male	Solid	Solid	Blood	1 week	13 weeks	9.69	7.61	−2.08	0.037

BM bone marrow.

^a^Refers to number of weeks after original diagnosis.

^b^Average methylation beta value increase across the 146 identified DMRs.

### Study 2

To investigate whether these DNA methylation changes are transient or maintained into adulthood, we examined the methylation patterns in DNA derived from blood samples from a different group of survivors of childhood/young adult cancer and compared with an age/sex matched control population. Adult childhood cancer survivors (CCS) (*n* = 32) were selected from the European Prospective Investigation into Cancer and Nutrition (EPIC) cohort, an ongoing multi-centre prospective cohort study with participating centres in ten European countries (further details on the cohort can be found elsewhere [17]). Cancer survivors diagnosed under 25 years old and who had had been diagnosed at least 10 year prior to EPIC recruitment (averaging 22 years post-diagnosis) were selected. This sample size was calculated to allow assessment of retention of methylation changes identified in study 1 (>99% power to identify the retention of the post-treatment signature identified in study 1 and >80% power to identify changes at the majority of individual DMRs (105/146; 72%) and was not designed to allow a genome-wide analysis in the adult CCS.

Specific treatment information was not available, so the analysis was restricted to tumour types in which all survivors would have been treated with chemotherapy and/or radiotherapy. Due to sample availability, we concentrated on malignant lymphoma and testicular cancer survivors (Table 2). Although specific treatments received by survivors were not known, a subset of the lymphoma survivors (*n* = 8) were treated before the use of chemotherapy became generally accepted for this disease (i.e., those diagnosed pre-1970). The 32 available cases included: Hodgkin lymphoma (*n* = 23), Non-Hodgkin lymphoma (*n* = 6), and testicular cancer (*n* = 3) diagnosed between 1958–1986 (Table 2). Blood samples were collected at EPIC study entry (1992–1999) and derived from centres in the UK (*n* = 13), Germany (*n* = 12), Italy (*n* = 2), Norway (*n* = 2), and Spain (*n* = 3). Study participants had provided informed consent and the EPIC study was approved by the relevant ethical review boards of each centre and from the International Agency for Research on Cancer.

**Table 2:** Summary of the adult CCS characteristics (study 2).

ID	Country	Sex	Cancer type	Age diagnosed (years)	Year diagnosed	Sample age (years)	Mortality	Age vital status^a^	Second cancer	Age second cancer	Average DMR methylation^b^	Age acceleration (years)^c^
1	Italy	F	HL^d^	21.95	1977	40.42	Alive	57.86	No	NA	0.07	7.26
2	UK	F	HL	24.32	1973	46.42	Alive	66.47	No	NA	0.052	3.67
3	UK	F	HL	24.12	1981	38.39	Alive	57.73	No	NA	0.116	7.93
4	UK	F	HL	18.33	1986	28.11	Dead	28.58	No	NA	0.13	1.99
5	UK	F	HL	16.7	1981	31.45	Alive	50.55	No	NA	0.084	9.26
6	Spain	M	HL	14.39	1969	41.21	Dead	43.39	No	NA	0.065	2.68
7	Italy	F	HL	22.31	1979	38.46	Alive	53.42	No	NA	0.112	−0.94
8	UK	F	NHL	10.91	1983	23.8	Alive	42.48	No	NA	0.082	0.38
9	Germany	M	NHL	17.49	1963	49.81	Alive	60.64	No	NA	0.022	1.66
10	Germany	M	HL	23.13	1972	46.42	Dead	48.4	Yes	48/	0.035	5.31
11	Norway	F	HL	24.17	1977	43.12	Dead	47.86	Yes	47/	0.049	3.59
12	UK	F	HL	22.86	1959	59.02	Alive	75.81	No	NA	0.053	−2.86
13	Norway	F	NHL	24.33	1976	44.21	Alive	55.38	No	NA	0.03	0.45
14	UK	M	Testicular	18.49	1970	43.6	Alive	60.02	No	NA	0.062	2.92
15	Germany	M	Testicular	24.94	1979	40.95	Alive	53.56	No	NA	0.024	−1.55
16	Germany	M	Testicular	24.7	1980	40.44	Alive	57.76	No	NA	0.027	1.64
17	UK	F	HL	24.4	1970	49.82	Dead	63.4	Yes	63/	0.075	6.55
18	Spain	M	NHL	18.99	1970	44.3	Dead	47.99	No	NA	0.072	6.09
19	Germany	F	HL	15.65	1976	35.52	Alive	47.73	Yes	47/	0.043	3.01
20	Germany	M	HL	18.42	1958	56.27	Alive	75.15	Yes	57/	−0.001	1.99
21	UK	F	HL	22.61	1980	38.52	Dead	53.69	Yes	48/52/54/	0.057	2.24
22	Germany	F	HL	17.85	1964	49.44	Alive	61.16	No	NA	0.053	1.28
23	Germany	F	HL	20.68	1974	42.09	Alive	59.7	Yes	43/	0.061	8.37
24	UK	F	HL	22.93	1977	41.48	Alive	58.98	No	NA	−0.005	0.29
25	Spain	M	HL	24.04	1979	40.08	Alive	51.26	No	NA	0.077	14.37
26	Germany	M	HL	24.43	1976	43.44	Alive	55.25	No	NA	0.042	2.29
27	UK	F	HL	22.74	1974	44.73	Alive	61.29	Yes	43/	0.042	1.02
28	UK	F	HL	19.79	1973	42.14	Alive	58.47	Yes	49/	0.055	5.63
29	Germany	F	NHL	14.7	1962	48.22	Alive	65.37	No	NA	0.062	1.05
30	UK	F	HL	18.62	1966	48.12	Alive	66.28	No	NA	0.031	3.03
31	Germany	F	NHL	15.94	1962	49.38	Alive	66.2	Yes	44/	0.065	3.88
32	Germany	F	HL	16.65	1976	35.85	Alive	46.64	No	NA	0.038	6.47

*HL* Hodgkin lymphoma, *NHL* non-Hodgkin lymphoma.

^a^Age vital status reflects age of death in deceased adult CCS.

^b^Average methylation beta value increase across the 146 identified DMRs.

^c^Age acceleration is skin-blood DNA methylation age relative to chronological age.

For study 2, a general population adult control dataset, with publicly available Illumina MethylationEPIC array data was identified from the GEO database. The control population were derived from the Michigan polybrominated biphenyl (PBB) research registry (http://pbbregistry.emory.edu/) [18] and comprised those with methylation data (*n* = 658) [19] with <1 ppb serum PBB, which is the threshold used to classify “no exposure” or “below detection level” in several studies [18, 20]. Controls were matched to the adult CCS cases (from EPIC) using the nearest neighbour algorithm with the “MatchIT” R package in a 9:1 ratio by age (±2 years) and sex (controls, *n* = 288; adult CCS, *n* = 32) (Table 3).

**Table 3:** Descriptive characteristics of adult CCS and controls (Study-2).

Adult CCS		Cases		Controls		
		*n*	%	*n*	%	*p*^a^
All		32		284		
Sex (female)		22	68.8	188	66.2	0.77
	HL		26	81.3		
Cancer site	Non-HL	3	9.4			
	Testicular	3	9.4			
		Mean (SD)	Range	Mean (SD)	Range	*p*^b^
Current age (years)		42.6 (7.21)	23.8–59.0	44.4 (6.76)	23.0–62.4	0.21
Time diagnosis to sample (years)		22.3 (7.21)	9.8–37.8	–	–	–

*SD* standard deviation, *HL* Hodgkin lymphoma.

^a^From *χ*^2^ test.

^b^From unpaired *T*-test.

### Genome-wide DNA methylation arrays

Bisulfite conversion was performed using the Zymo EZ-96 DNA methylation kit (Zymo Research). Genome-wide DNA methylation was quantified in all samples using the Infinium MethylationEPIC BeadChip microarray, which evaluates genome-wide CpG methylation at over 850,000 sites and carried out at the Wellcome Trust Clinical Research Facility, University of Edinburgh (Edinburgh, UK). Raw methylation data from MethylationEPIC arrays for all test and control samples were processed using the minfi Bioconductor package version 1.28.4 in R studio version 3.5.3. Sample quality was confirmed (examining array control probes and gender discordance) for all samples. Samples which failed quality control were excluded (*n* = 4 adult control samples). The single-sample (ssNoob) method was used for normalisation [21]. Cell composition was estimated using the Houseman method [22]. Probes with a detection *p*-value >0.01 and cross-reactive probes (i.e., probes which cross-hybridise between autosomes and sex chromosomes) [23] were removed. After processing, 820,139 probes remained for the childhood paired samples (*n* = 820,134 of these passed quality control in the adult samples). Methylation values were transformed to *β* values, which range from 0 (0% methylation) to 1 (100% methylation), representing methylation intensity [24].

### Identification of differential methylation in CCS

For Study 1, differentially methylated regions (DMRs) were identified using the DMRcate R package with the default settings [25]. Differentially methylated positions (DMPs) were identified in late vs. early remission using the Limma R package (lmFit function). Differential DNA methylation was summarised according to the direction of change (from early to late remission), and all analyses of differentially methylated loci were corrected for multiple testing using false discovery rate correction (FDR). Correlation values between DMRs were calculated using Pearson’s correlation for each cancer type and averages compared.

CpG sites and DMRs were annotated with their genomic position, nearest gene and relation to gene/CpG island. Differentially methylated CpG loci were annotated using the HumanMethylationEPIC array and UCSC annotations for genes, genomic regions (i.e., gene body, untranslated regions, transcriptional start site (TSS), exon) and island positions (island, shore, open-sea). Regional enrichment for identified loci compared to overall distribution on the EPIC array was assessed using chi squared (*χ*²) tests.

For study 2, we examined methylation differences between adult CCS and controls samples, focusing only on the DMRs and DMPs (with >5% absolute change in methylation) identified in study 1. The differences in DNA methylation between adult CCS cases and controls at individual CpG sites and DMRs were determined. Statistical differences were examined using *t*-tests. A single differentially methylated score was calculated for each survivor by averaging the changes across all retained DMRs. DMRs were defined as being replicated in adult survivors if they were statistically significantly different from the control population, exhibited the same direction of change as seen following exposure to treatment, and had an absolute difference in methylation of at least 50% of the change identified in the post-treatment samples in study 1. Annotation of CpG sites and the analysis of genomic positions (relative to genes and islands) was done as above, and distributions of the original DMRs and the retained DMRs were compared using chi squared (*χ*²) tests.

Linear regression was used to determine if there were differences in methylation at individual DMRs (dependent variable) between cases and controls (independent variable) adjusted for age, sex, and white blood cell proportions. *p*-values were adjusted for multiple testing using FDR correction. Stratified linear regression analyses were similarly carried out in lymphoma cases to distinguish any notable differences in DMR methylation between cases diagnosed before or after the introduction of chemotherapy as a standard treatment for lymphoma (i.e., patients diagnosed before or after 1970) [26].

### Epigenetic clock analysis

Epigenetic age using the skin-blood clock (hereinafter referred to as epigenetic age) was estimated for all samples, and surrogate DNA methylation biomarkers (study 2) using Horvath’s online estimator (available at https://dnamage.genetics.ucla.edu) [27, 28]. Although the clock was developed using 450 K array data, EPIC array data has been shown to accurately estimate DNA methylation age regardless of the missing CpG sites (*n* = 19) [29]. Epigenetic age before and after treatment (study 1) was compared using a paired *t*-test, and by cancer site using two sample *t*-test (study 1) or ANOVA (study 2).

In study 2, to further understand markers of late effects in CCS we examined surrogate DNA methylation biomarkers known to be associated with chronic diseases, such as PhenoAge which is associated with health span [30], or GrimAge, associated with mortality [31]. Correlations between DMRs and epigenetic age measures were examined using Pearson correlation, and differences in epigenetic age acceleration markers (dependent variable, listed in Supplementary Table 10) between adult CCS and controls (independent variable) using linear regression adjusted for age and sex.

### Long-term health outcomes in adult CCS

Long-term follow up data for the adult CCS was collected as part of the EPIC study, including survival and development of second primary cancers (average follow-up 16.8 years). Baseline characteristics and DNA surrogate markers were compared between alive vs. deceased, and between those who had second cancers vs. those who had not, using *χ*² tests for categorical variables and two-sample *t*-tests for continuous variables. Associations between DMR methylation or surrogate DNA methylation biomarkers and vital status/second cancer were examined using linear regression adjusted for age and sex.

## Results

### Exposure to cancer therapy results in aberrant genome wide DNA methylation patterns in normal cells

**Fig. 1:**
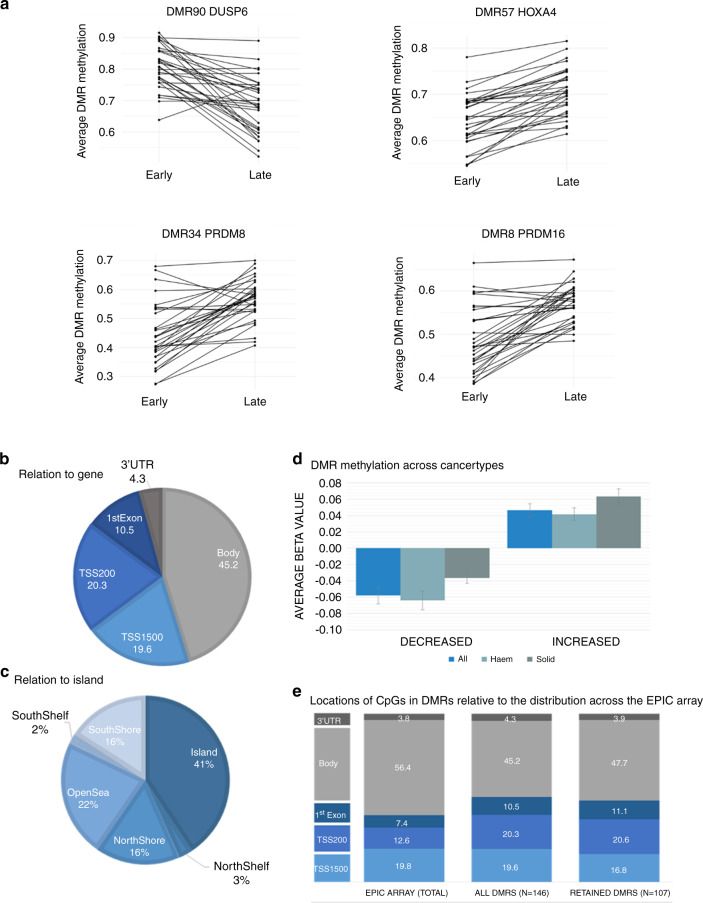
Differentially methylated regions (DMR) identified in late remission samples from childhood cancer patients (*n* = 32). **A** Examples of altered methylation between the early and ate remission samples in the 32 paired sample sets at four of the identified DMRs (DMR and nearest gene, as indicated). Summary of the locations of DMRs (*n* = 146) relative to **B** genes and **C** CpG islands. **D** The average change in DMR methylation (beta value) in the sample set as a whole (all, *n* = 32) and for the haematological (Haem, *n* = 25) and solid tumours (solid, *n* = 7) specifically. Error bars represent SEM. **E** The locations of the CpGs sites in the identified DMRs (*n* = 1045) in the full set of DMRs (All DMRs) and the subset of DMRs retained in the adult CCS (Retained DMRs) relative to the distribution of CpGs across the EPIC array. 18 DMRs had no associated gene and are therefore not represented.

To investigate the possibility that anti-cancer therapy is associated with altered DNA methylation in normal tissues, we assessed genome-wide methylation patterns in paired blood/bone marrow samples in a cohort of 32 childhood cancer patients undergoing multi-agent chemotherapy. Average time between paired samples was 43.5 weeks (Table 1). Cell type distribution was similar before and after exposure to cancer therapy, although there was a borderline significant drop in B-lymphocytes seen in both haematological and solid tumour patients (Supplementary Table 2). Genome-wide methylation analysis identified 146 differentially methylated regions (DMRs) which were consistently changed in post-treatment samples across the study population. The overwhelming majority (140 DMRS) of these exhibited increased methylation (examples in Fig. 1A, full details in Supplementary Table 3).

The identified DMRs exhibited similar patterns of altered DNA methylation across all tumour types analysed. All 146 DMRs exhibited altered methylation in both solid and haematological malignancies, with 99.3% (145/146) exhibiting methylation changes in the same direction (Supplementary Tables 4 and 5). Differences between tumour types were not statistically significant (Table 4, ANOVA *p* = 0.11), although sample size was small. Alterations in methylation were slightly higher in solid tumour patients (6.2% vs. 4.2%, Fig. 1D) with the majority of DMRs (74.0%, 108/146 DMRs) exhibiting greater absolute methylation changes in this group. This may be a consequence of the initial sample from solid tumours patients being truly pre-treatment, while for haematological cancers the first sample was at initial remission, after several weeks of therapy (Table 1).

**Table 4:** Average DMR methylation change post-treatment (n = 146) by cancer type in childhood cancer patients.

Cancer type	*n*	Mean change Beta value^a^	SD	Min	Median	Max
All samples	32	0.047	0.029	−0.011	0.043	0.104
Haematological^b^	25	0.042	0.027	−0.011	0.039	0.101
Solid^b^	7	0.062	0.032	0.016	0.055	0.104

*SD* standard deviation, *min* minimum, *max* maximum.

^a^Change in beta value (post-treatment vs. pre-treatment sample), averaged across the 146 DMRs.

^b^Haematological cancers include ALL (*n* = 22), AML (*n* = 2) and lymphoma (*n* = 1), while solid tumours include neuroblastoma (*n* = 5), osteosarcoma (*n* = 1) and Wilms’ tumour (*n* = 1), derived from male (*n* = 20) and female (*n* = 12) patients. Differences between solid and haematological cancers were not statistically significant (ANOVA *p* = 0.11).

**Fig. 2:**
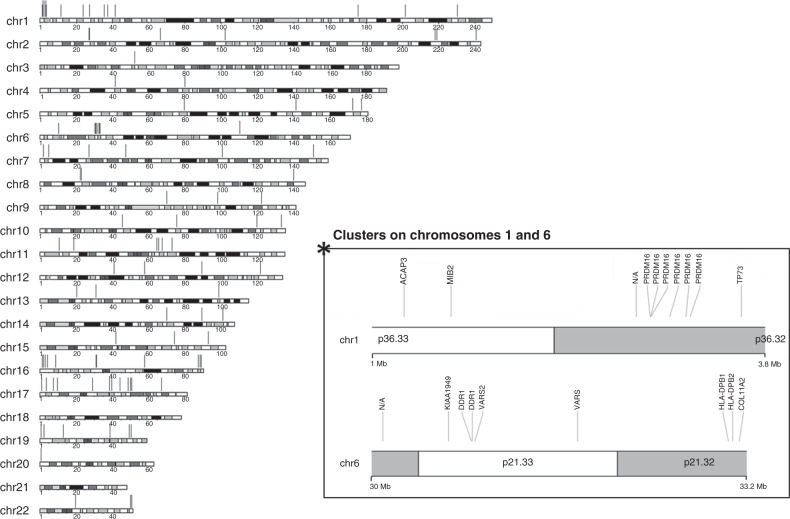
Chromosomal locations of the DMRs identified in later remission samples from childhood cancer patients (*n* = 32). Genomic positions of the DMRs across chromosomes 1–22 are illustrated by red vertical lines. The DMRs were found spread across all 22 autosomic chromosomes, but with two pronounced clusters on chromosome 1 and 6. The X/Y sex chromosomes are not shown as these were excluded from the differential methylation analysis. The DMR clusters on chromosomes 1 and 6 are shown in more detail on the inset. Locations of specific genes are indicated on the chromosome cluster panel. Sizes on chromosome bands are in megabases (Mb). N/A denotes no associated gene. Chr chromosome, Mb megabase.

Genomic locations of DMRs relative to gene locations and CpG islands are shown in Fig. 1. 50.5% of the CpG sites within the identified DMRs were located in, or proximal to, gene promoter regions (i.e., TSS (transcriptional start site) and 1st exon, Fig. 1B), with the remaining DMRs identified at multiple positions throughout genes. A subset were not clearly associated with any expressed sequence (*n* = 18). DMRs were enriched for sites within TSS200 (200 bp upstream of the TSS) and the 1st exon compared to sites on the EPIC array as a whole (Fig. 1E, *χ*² *p* < 0.001, Supplementary Table 6) and were also more likely to be in islands and North and South shores, (*χ*² *p* < 0.001, Supplementary Table 7, shores defined as the 2 Kb immediately before and after a CpG island). The DMRs were distributed throughout the genome (Fig. 2), with two apparent clusters on chromosome 1 (p36.33–p36.32; cluster of ten DMRs (*n* = 47 CpG sites, 2.4 Mb region), with six of these mapping to the *PRDM16* gene) and chromosome 6 (p21.33–32; a cluster of eight DMRs (*n* = 128 CpG sites, 3.1 Mb region) mapping to the HLA region).

### Altered DNA methylation is replicated in adult survivors

**Fig. 3:**
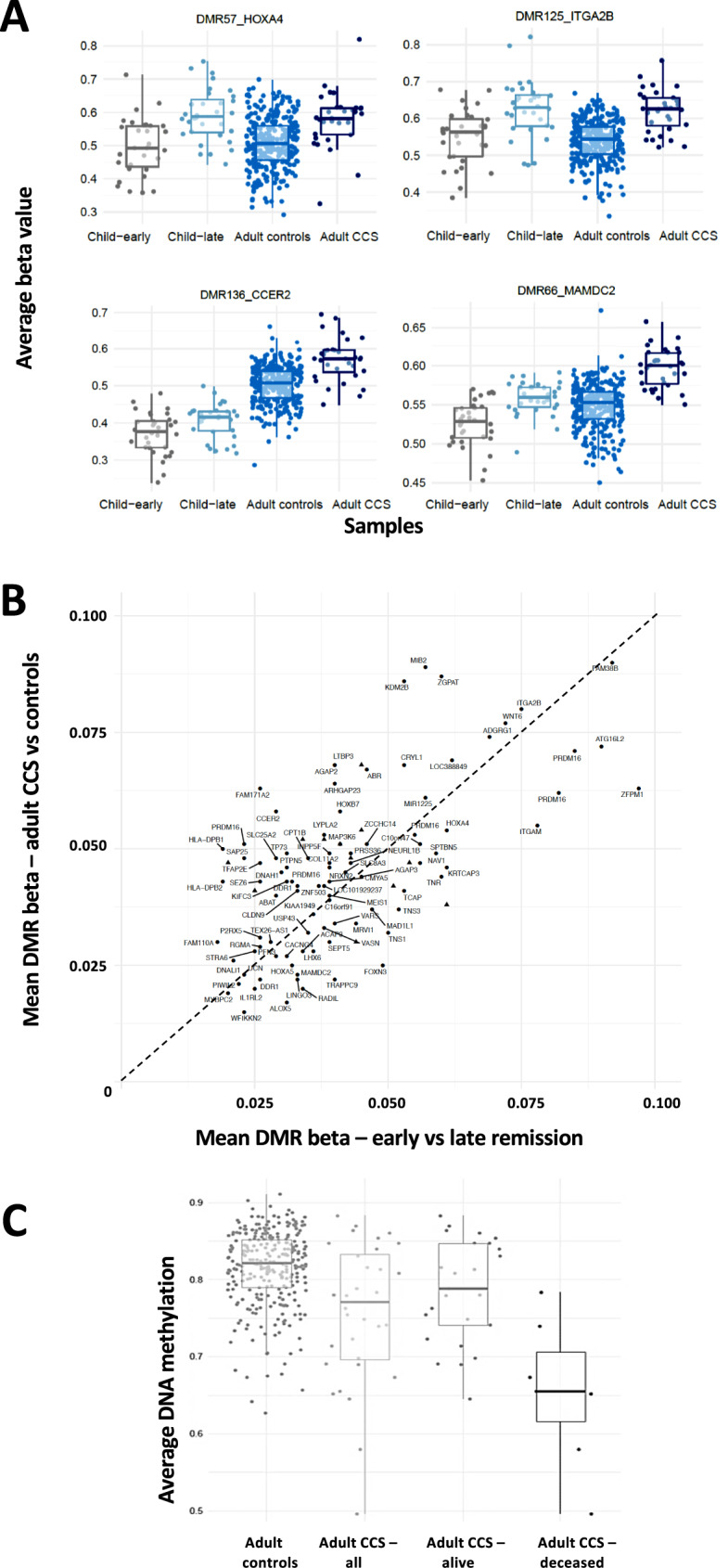
DMRs are retained in adult CCS and associate with premature mortality. **A** Beta values are shown for four representative DMRs to illustrate the relative differences between early and late remission in the childhood cancer patients (*n* = 32) compared with adult controls (*n* = 284) and adult CCS (*n* = 32). Box plots show relative distribution of methylation at the specific DMRs in individual groups and dots illustrate specific values in individual samples. Top two panels (DMRs at the HOXA4 and ITGA2B loci) show similar level of methylation in both control sample sets with similar increased methylation in late remission samples and adult CCS. Bottom two panels (DMRS at the CCER2 and MAMDC2 loci) show examples with time dependent increased methylation in the two control sample sets, but with similar sized increases versus their respective controls in late remission samples and adult CCS**. B** Average beta change in DMRs (*n* = 107) in childhood cancer patients (early vs. late remission, *n* = 32) and adult CCS (*n* = 32; compared to adult controls, *n* = 284). Nearest gene to each DMR is indicated for each point. A triangle indicates DMR has no associated gene. DMRs above the dotted line had increased DNA methylation in adult CCS relative to changes originally identified in post-treatment childhood cancer patients, while those below the line had lower methylation in CCS. **C** DNA methylation at DMR90 (*DUSP6* locus) is associated with mortality in adult CCS (*n* = 32: alive *n* = 25, deceased *n* = 7). Average beta methylation at DMR90 is shown for adult CCS (*n* = 32) and adult controls (*n* = 284), as indicated. Adjusted linear regression *p*-value for the difference between all adult cases and controls (a) and for alive adult CCS vs. deceased adult CCS (b) are shown.

Methylation patterns at the 146 DMRs, identified in study-1, were assessed in the adult-aged CCS relative to the age-matched control population (study-2) to determine if the differences in methylation identified post-therapy were replicated in adult survivors. A DMR was regarded as replicated if the identical region exhibited a significant difference between the adult survivors and the controls, had the same direction of change and an absolute difference of at least 50% of the size seen in study 1. By focusing on the 146 DMRs, the power of the analysis is enhanced, and identification of false positives reduced (compared with a genome-wide analysis using this number of samples). The average duration between the cancer diagnosis and DNA sampling in the adult survivor cohort was 22.3 years (Table 2). It is possible that replication of apparently altered methylation could be secondary to retention of the reduced B lymphocyte fraction observed post-treatment in study-1. However, adult CCS did not show any reduction in B lymphocyte fraction versus the control population (Supplementary Table 2). A small but statistically significant reduction in CD4+ T-cells was observed in the adult CCS (0.12 vs. 0.14, *p* = 0.01) and so all subsequent analysis was adjusted for differences in cell type distribution (in addition to age and sex). The majority of DMRs were replicated in this sample set (73% (107/146), with adult CCS exhibiting statistically significant differences in methylation versus the non-CCS controls (examples in Fig. 3A, top 20 DMRs in Table 5, full details in Supplementary Table 3 and Supplementary Fig. 1). Furthermore, the methylation levels at the 107 replicated DMRs in the adult CCS (vs. the adult controls) closely mirrored the extent of altered methylation observed in post-treatment (late remission) childhood cancer patients (i.e., they cluster around the dotted blue line representing equal methylation changes in both groups in Fig. 3B), implying that the altered DNA methylation identified in post-treatment childhood cancer patients may be largely stable and retained many years after treatment has ceased. The levels of differential methylation at specific CpG sites across the DMRs were also similar in adult survivors (versus controls) as had been seen in study-1 in the post-treatment samples (Supplementary Fig. 2). There were no significant differences in the genomic or island locations of CpGs in the replicated DMRs compared to the non-replicated DMRs (Supplementary Tables 6 and 7). In contrast to the frequent differential methylation at DMRs, only 47.7% of non-DMR associated DMPs were differentially methylated in CCS relative to controls (Supplementary Table 8).

**Table 5:** The 20 retained DMRs with largest absolute difference between CCS and control population.

DMR	Nearest Gene	Difference in beta value	95% CI	pFDR
DMR34	*PRDM8*	0.091	(0.065,0.117)	<0.001
DMR92	*CRYL1*	0.084	(0.062, 0.105)	<0.001
DMR91	*KDM2B*	0.079	(0.057, 0.102)	<0.001
DMR132	*FAM38B*	0.076	(0.052, 0.101)	<0.001
DMR2	*MIB2*	0.075	(0.050, 0.100)	<0.001
DMR27	*WNT6*	0.075	(0.053, 0.096)	<0.001
DMR125	*ITGA2B*	0.071	(0.055, 0.087)	<0.001
DMR8	*PRDM16*	0.066	(0.056, 0.076)	<0.001
DMR140	*ZGPAT, LIME1*	0.066	(0.055, 0.077)	<0.001
DMR4	*FLJ42875, PRDM16*	0.065	(0.057, 0.074)	<0.001
DMR134	*LINGO3*	0.062	(0.031, 0.092)	<0.001
DMR6	*PRDM16*	0.061	(0.046, 0.076)	<0.001
DMR21	*KRTCAP3, NRBP1*	0.061	(0.021, 0.101)	0.003
DMR114	*ZFPM1*	0.061	(0.045, 0.076)	<0.001
DMR90	*DUSP6*	-0.061	(-0.079, -0.044)	<0.001
DMR75	N/A	0.06	(0.046, 0.075)	<0.001
DMR87	*ATG16L2*	0.06	(0.029, 0.091)	<0.001
DMR18	*NAV1*	0.059	(0.030, 0.089)	<0.001
DMR143	*LOC388849*	0.059	(0.042, 0.076)	<0.001
DMR57	*HOXA4*	0.058	(0.031, 0.085)	<0.001

*CI* confidence interval, *FDR* false discovery rate.

Models are adjusted for age, sex and cell proportions. Estimates reflect the difference in DNA methylation in cases relative to controls.

A subset of the lymphoma survivors (*n* = 8) would almost certainly have been predominantly treated with radiotherapy (as they were diagnosed pre-1970 before the use of chemotherapy became accepted practice in this disease). They exhibited similar altered methylation across the DMRs. The extent of altered methylation changes was generally lower in these individuals, although this difference was not statistically significant (Supplementary Table 9).

### Accelerated epigenetic ageing in adult childhood cancer survivors

To investigate if exposure to cancer therapy was associated with accelerated epigenetic ageing, we assessed the above sample sets using the skin-blood methylation clock [28]. In the childhood cancer patients undergoing therapy (study-1) there was no evidence of accelerated ageing, and indeed the samples at the end of therapy exhibited a reduction in apparent epigenetic age (average reduced ageing 1.1 years, paired *t*-test *p* = 0.003, Table 1). In contrast, the adult survivor population exhibited clear age acceleration when compared to the control cohort (average age acceleration +5.5 years (95% CI 4.32, 6.61, *p*_FDR_ < 0.001, Supplementary Table 10). There was no significant correlation between the age acceleration and average DMR change in individual adult CCS (*r* = 0.31, *p* = 0.09, Table 2) and the CpG sites within the DMRs were not significant associated with age in the control population (Supplementary Fig. 3), suggesting that these two measures of epigenetic change were largely independent.

In contrast to the skin-blood clock, the GrimAge methylation clock (designed specifically to predict risk of mortality [31]), did not show any significant age acceleration. Significant differences in surrogate DNA methylation biomarkers were observed in the adult CCS population (including: higher ADM, B2M, Cystatin C, Leptin, PAI1, TIMP1, and decreased telomere length, Supplementary Table 10) and in each case these are altered in the direction previously associated with higher likelihood of chronic disease development.

### Differential DNA methylation and later-life health outcomes

Adult CCS exhibit higher rates of second cancers and early mortality. Consistent with this, one-third of the adult CCS in this study developed second primary malignancies (10/32; 31%) and one-fifth were deceased (7/32; 22%) at a relatively young age (median age 48, range 23–63 years, median follow-up 15.8 years). CCS who experienced a second malignancy or had died were not demographically different from other survivors (Supplementary Table 11). Second cancers were not associated with DMR methylation or epigenetic age acceleration. However, there was a trend towards higher average DMR methylation in the participants that had died at follow-up (6.9% vs. 5.1%) and analysis of the individual DMRs, identified DMR90 (*DUSP6*) as significantly associated with mortality, even after correction for multiple testing (*p*_FDR_ < 0.001, Fig. 3C and Supplementary Table 12). In addition, there was a significant age-acceleration (8.8 years, *p* < 0.001), specifically for the GrimAge clock, in deceased versus surviving members of the CCS group (Supplementary Table 13). This provides initial potential evidence suggesting that altered DNA methylation may predict certain important health outcomes in the CCS population.

## Discussion

Childhood cancer patients exhibit greatly increased risk of adverse health outcomes in later life [3]. We hypothesised that changes in DNA methylation could contribute to increased levels of chronic disease in CCS. Here we demonstrate significantly altered genome-wide DNA methylation in childhood cancer patients following anti-cancer therapy. The majority of the DMRs are also evident in adult CCS, 10–38 years after diagnosis. These changes were consistent across different cancer types and in patients who were likely to have undergone different treatments (different chemotherapy regimens and/or radiotherapy). While there was some overlap in the treatments given to the patients whose samples were used in study-1 (for example all had regimens that included a topoisomerase inhibitor [16]), there would have been little overlap with the treatment given to patients included in study-2, which included a subset of patients likely to have been treated with radiotherapy alone. Thus, the identified alterations in DMR methylation are likely to be largely treatment type independent. However, as the study was not designed (or powered) to identify changes associated with specific drug treatments, the possibility of such treatment-specific changes in methylation cannot be excluded.

Childhood cancer patients (study-1) did not show evidence of age acceleration in late compared to early samples, however, age acceleration was clearly evident in the adult CCS population (study-2). As the DMR methylation only weakly correlated with the accelerated epigenetic ageing, this suggests the possibility that CCS exhibit two different epigenetic abnormalities; a large set of differentially methylated genes that are acutely associated with therapy and then retained long-term (for at least several decades), and alterations in DNA methylation that lead to accelerated epigenetic ageing that arise in survivors post- treatment. Our current study provides initial evidence that these methylation changes may correlate to subsequent patient outcomes, but larger, ideally longitudinal, studies of adult CCS will be required to more clearly determine the extent to which these epigenetic changes can be used to predict survival or chronic disease development in the CCS population.

A potential explanation for the consistent alterations in DNA methylation following exposure to different therapies is that these are reflective of selection for subsets of normal cells that are more able to survive exposure to cytotoxic treatments/cellular stress. Some of the genes associated with the DMRs we identified have previously been described as having altered methylation following exposure to chemotherapy. For example, we have previously determined that exposure to chemotherapy leads to increased levels of DNA methylation at the *HOXA4* locus in remission samples from both adult and childhood ALL [15], a finding that was replicated here. In addition, we have demonstrated that treatment of chronic lymphocytic leukaemia patients, results in selection for altered methylation of a number of genes, including hypermethylation of *HOXA4*, in the leukaemia cells [32]. Restoration of HOXA4 expression re-sensitises CLL cells to multiple therapeutic agents, identifying a direct role for HOXA4 in control of sensitivity to chemotherapeutic agents with different mechanisms of action [32]. Similarly, exposure of normal cells to anti-cancer agents may select out sub-populations of cells with methylation patterns associated with increased resistance to cellular damage/stress resulting in similar methylation changes in response to different cytotoxic agents.

We identified a set of DMRs exhibiting consistent changes post treatment, although the absolute magnitude of methylation changes varied between patients. This study was not powered to identify the underlying cause of this variation, which could reflect inter-individual differences, different therapies or different tumour types. Larger differences in altered methylation were observed in solid tumours vs. haematological cancers. However, the solid tumours also differed according to the timing of the first sample taken (generally near diagnosis for the solid tumours, but after remission for the haematological malignancies). Furthermore, when the analysis was restricted, post hoc, to just ALL samples, a correlation was identified between the timing of the initial sample and the overall level of methylation change (data not shown) suggesting that the timing of the initial sample may be an important determinant of the absolute size of methylation changes at the DMRs. However, changes across the same set of DMRs were seen regardless of the timing of the initial sample.

We identified differential methylation of genes that have been related to cancers such as lymphoma (*WNT6, TP73,* and *CD37*) [33, 34], leukaemia (*MEIS1, HOXA4*, and *HOXA5*) [35, 36] or neuroblastoma (*TP73*) [37], as well as genes related to cardiovascular diseases (*UCN, PRDM16, TCAP, ALOX5*) [38–41]. This may suggest that therapy-induced DNA methylation within these loci could directly contribute to some of the key life-threatening late health effects in CCS, such as secondary malignancies or cardiovascular disease. Future studies will be required to directly assess the impact of the identified methylation changes on expression of the linked genes. As the methylation changes are in the range of 2–13%, fine-tuned analysis at the single cell level may be more insightful than examining effects on gene expression in bulk tissue. In addition, functional analysis of candidate genes potentially involved in late health effects will be necessary to examine potential causal links between altered DNA methylation and disease in CCS.

The hypothesis that methylation changes induced by cancer treatments could impact long-term health was first proposed by Lyon et al. [42]. However, subsequent experimental testing of this hypothesis has been limited. This is the first study to examine acute changes in methylation following chemotherapy and whether methylation at the same loci also differed in adult cancer survivors. A small number of previous studies have looked specifically at DNA methylation in survivor populations. These have primarily concentrated on epigenetic ageing, and consistent with this study, have identified accelerated ageing in cancer survivors [43–46]. A recent study by Song et al. [47] also performed an epigenome-wide association study (EWAS) to identify specific CpG sites exhibiting differential methylation in patients given specific therapies or combinations of therapies. They identified 935 CpG sites associated with individual treatments and 224 CpG sites associated with exposure to drug combinations. Both sets of sites exhibited highly significant overlap with the CpG sites identified in this study, especially for those associated with drug combinations (36/224, 16.1% overlapping, *p* < 1 × 10^−50^ (Fisher exact test), with all differences at matching CpG sites altered in the same direction), further suggesting that these methylation changes are consistent consequences of exposure to highly toxic anti-cancer therapies.

Of particular interest was the cluster of DMRs identified within the *PRDM16* gene. As well as being linked to cardiovascular disease, PRDM16 deficiency has also been associated with the development of fibrosis, which is linked to multiple age-associated chronic diseases [48] and *PRDM16* methylation has been linked to obesity [49]. In addition, PRDM16 has been identified as a key controller of hematopoietic stem cells (HSC), where it functions to suppress HSC proliferation [50]. Thus, alterations in the methylation of this gene may be related to the requirement to repopulate the hematopoietic compartment after exposure to therapy, which requires downregulation of PRMD16 expression. Potentially, methylation changes associated with the downregulation of PRDM16 expression may lead to stabilisation of the reduced PRDM16 expression and consequent long-term tissue dysfunction.

Although the study was not powered to assess the association between the DNA methylation changes and clinical outcome, the results provide some initial evidence that the extent of altered methylation may act as a marker for key clinical endpoints. Average DMR methylation was associated with longevity, and methylation at one specific DMR (at the *DUSP6* locus) was statistically significant, even after correction for multiple tests. Larger studies will be required to properly assess the link between altered DNA methylation and specific chronic illnesses that are increased in CCS.

A strength of this study is the multi-tiered approach which utilised two datasets of CCS with epigenetic data and also included long-term follow-up data for adult CCS, as well as survival and clinical outcomes. As with all observational epidemiological studies, there is the possibility of residual confounding from unmeasured factors, such as lifestyle. A limitation when examining rare diseases such as childhood cancers is the small sample sizes. In this study, the number of adult CCS included in study-2 was powered to allow confirmation of the replication of methylation changes, which was very clearly detected, but was not sufficient to assess the association of altered DNA methylation in the CCS population with specific chronic health conditions. Larger prospective studies are required to more rigorously assess the link between altered DNA methylation and health outcomes, focusing particularly on the specific chronic illnesses for which CCS have increased risk.

Limitations of this study included the heterogeneity of the initial samples in study 1 (tumour types and therapies) and the relatively small sample size. This study did not aim to identify methylation changes induced in a drug specific fashion, however these changes may be differentiated in future studies with greater statistical power. As childhood cancer patients are typically treated with complex multi-agent approaches, we are now investigating the use of mouse models to more directly assess drug-specific methylation changes. This will also help determine whether any methylation changes could be induced by the disease itself, independent of treatment. Similarly, there was a lack of overlap between the cancer types included in study 2 with study 1, and the specific anti-cancer therapies used to treat the original cancers in study 2 participants were not known. While the overlapping methylation differences across the two diverse studies emphasises the general applicability of the loci that were replicated, it remains possible that some of the unreplicated DMRs may be more specific for the cancer therapies in study 1. Future research in larger series would be valuable to identify methylation changes associated with specific treatment protocols and specific cancer types. It is possible that the results of this study could also have been impacted by other environmental factors which can influence the epigenome [51]. However, using a within-patient design in study 1, and then concentrating on replicating these specific methylation changes in study 2, will have significantly mitigated this risk compared to non-directed EWAS studies of cancer survivor cohorts. We also identified no significant overlap between methylation changes associated with specific environmental factors (e.g., ageing methylation (Supplementary Fig. 3) or smoking, where we identified no statistically significant overlap between the DMRs identified in this study with CpG sites identified in the meta-analysis of Joubert et al. [52]. However, we cannot completely disregard the influence of other unmeasured environmental factors.

The findings of this study may have potential clinical utility. A key finding is the similarity in methylation changes seen between childhood cancer patients/survivors regardless of cancer type, what treatment they received or when they were treated. This suggests there may be a common set of methylation changes that are present across many childhood cancer patients following exposure to cytotoxic agents. These would therefore be widely applicable for assessing normal cell toxicity and potential long-term health effects across childhood cancer patients. Given that all specific types of childhood cancer are rare, this potential applicability to multiple cancer types would be a significant advantage of these methylation changes as prospective clinical markers. For instance, if the identified methylation changes are shown to be predictive of specific late health effects, these could be utilised in precision medicine approaches to identify high-risk individuals and to direct follow up care. In addition, since increased methylation at DMRs can be measured during treatment, this could potentially be used to evaluate ongoing trials of childhood cancer patients to assess whether new treatment protocols or dose de-escalation studies result in lower level of epigenetic damage.

Overall, these findings suggest that anti-cancer treatment in childhood cancer patients may induce a consistent set of DNA methylation changes, that occur largely independent of tumour type or specific treatment, which are retained in adult CCS and may be associated with adverse health outcomes. These findings offer significant scope for utilising multiple methylation markers (DMRs, combined or separately, and epigenetic age acceleration) in the development of risk prediction tools for CCS.

## Supplementary information

Supplementary files

Reproducibility checklist

## Data Availability

All array data used in this study is available on the NCBI GEO database. The array data generated as part of study-1 and study-2 have been submitted to GEO (accession number: GSE162560) and the control data used in study-2 had previously been deposited in GEO (accession number: GSE116339).
