# Supplementary material for: Anti-cancer therapy is associated with long-term epigenomic changes in childhood cancer survivors
Source: Br J Cancer. 2022 Mar 30;127(2):288–300. doi: 10.1038/s41416-022-01792-9 (PMC9296636; doi:10.1038/s41416-022-01792-9)
Supplement: Supplementary file 1 — Supplementary files [file 41416_2022_1792_MOESM1_ESM.docx]

**Supplementary Figures**

**Supplementary figure 1**

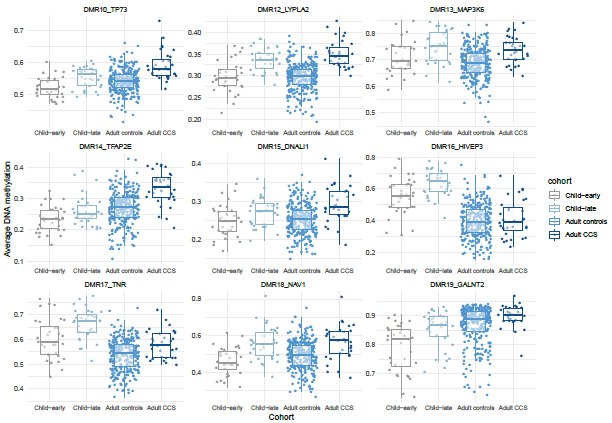


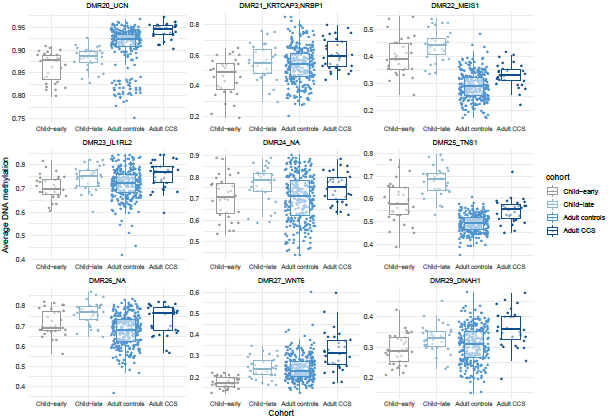

#
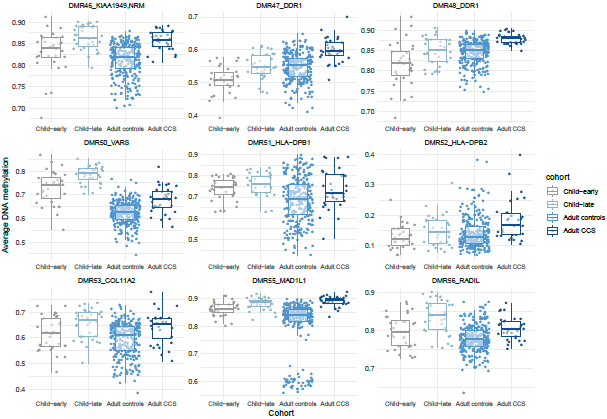


#
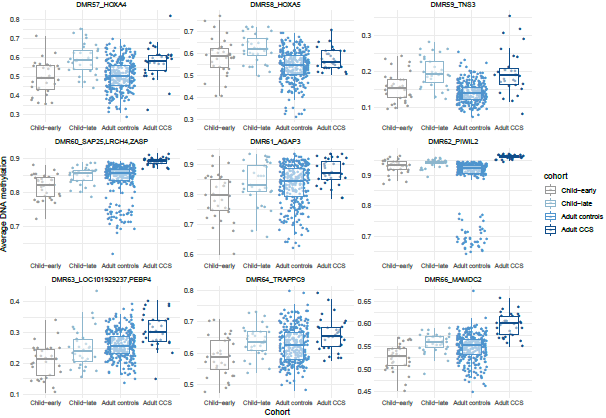


#
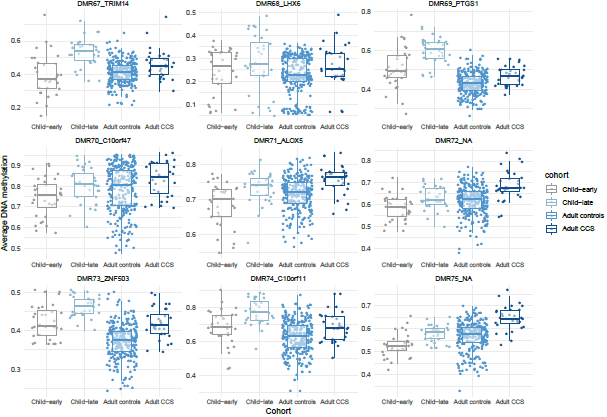


#
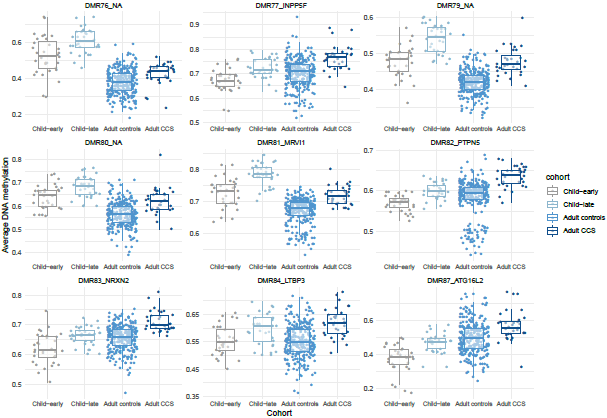


#
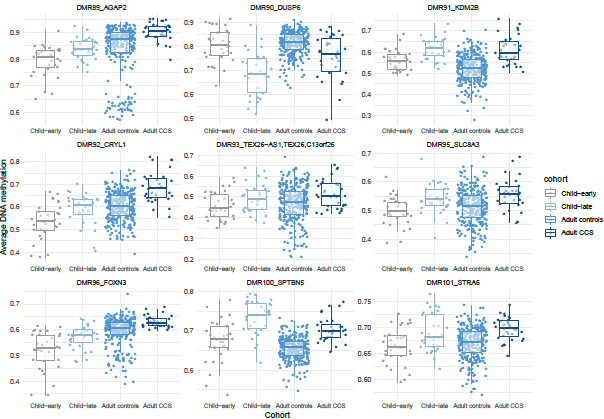


#
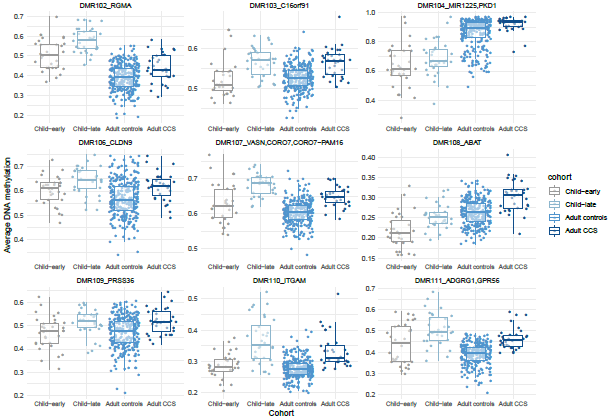


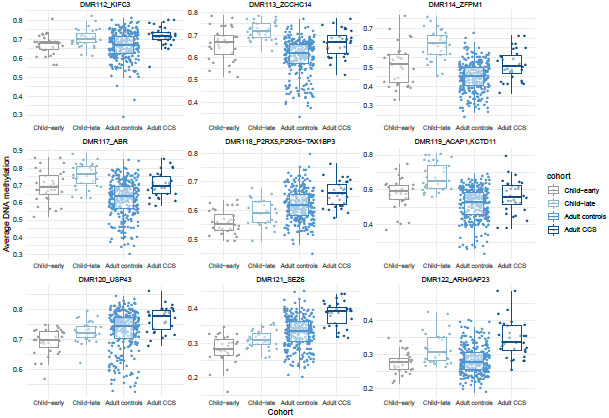


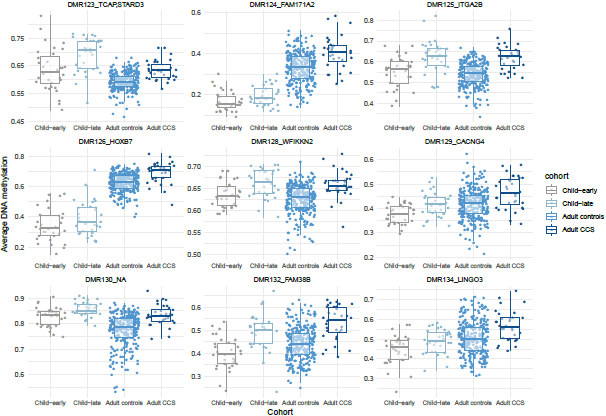


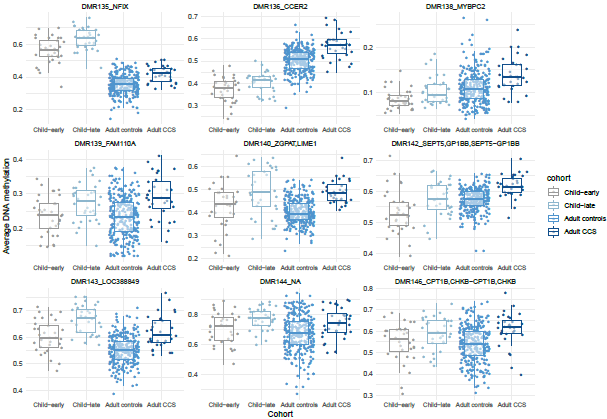


# **Supplementary Figure 1 – Boxplots of methylation levels at all retained DMRs in study 2**. Methylation levels for all samples in both study 1 and study 2 are expressed in beta values. The specific DMRs are listed above each plot (DMR number and nearest gene (NA, indicates that no gene was present within 20Kb of the DMR)).

**Supplementary Figure 2**


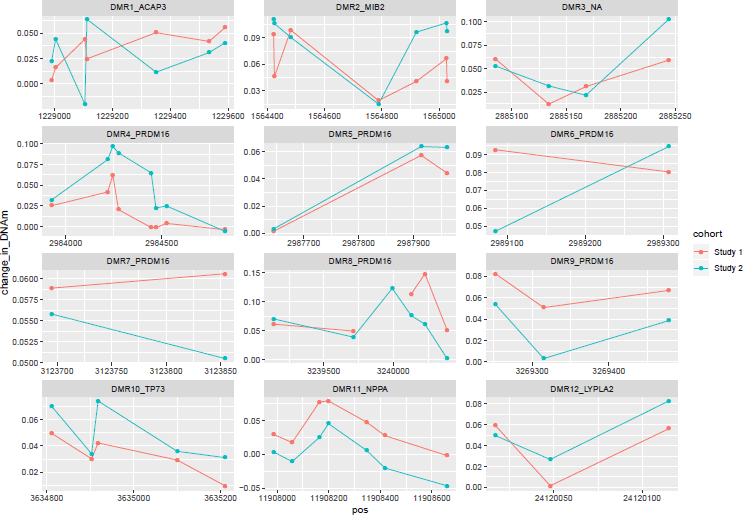


#
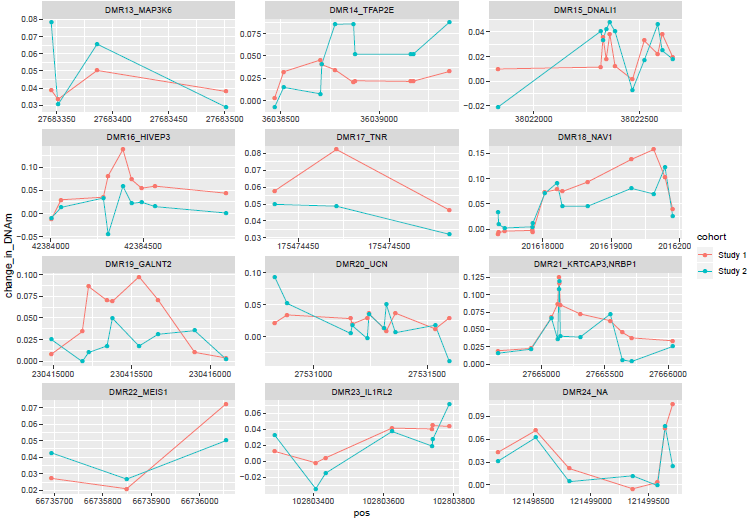


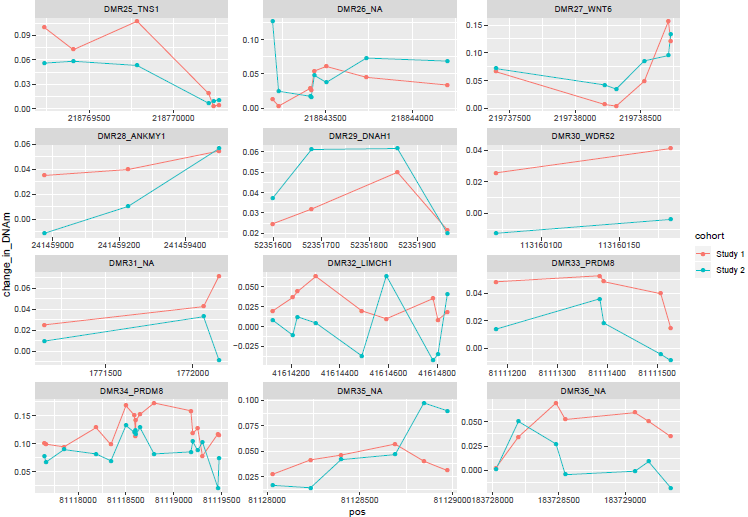


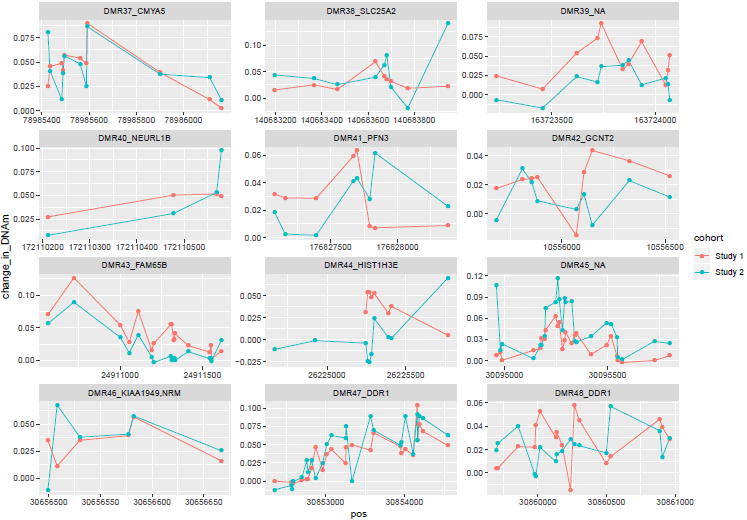


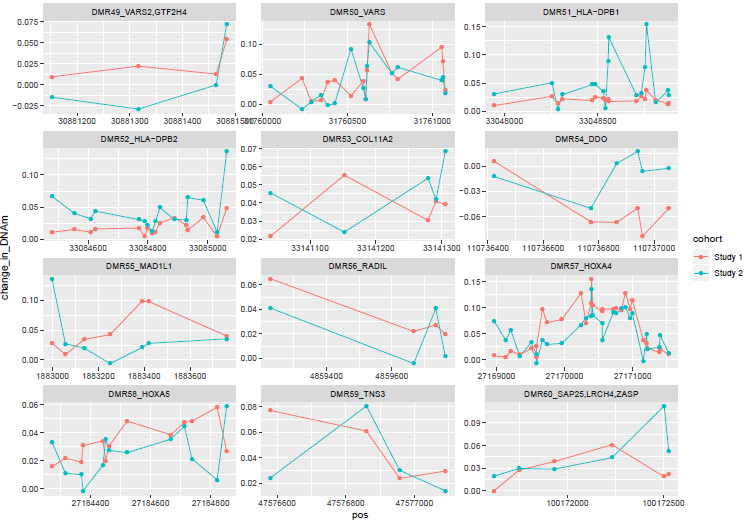


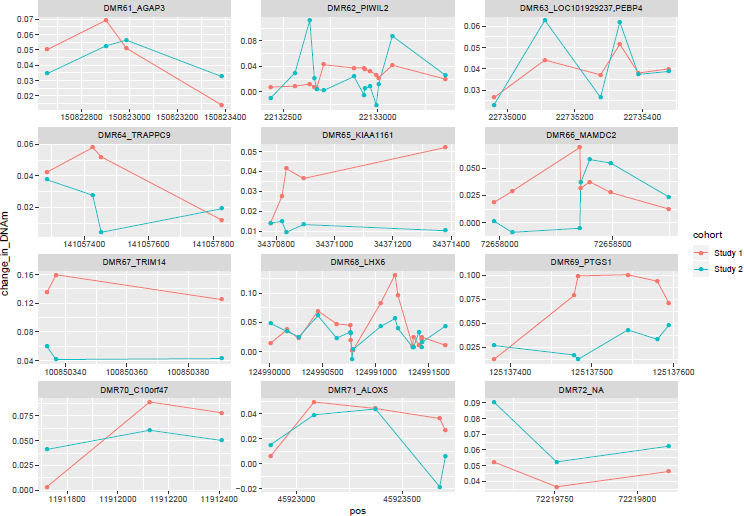


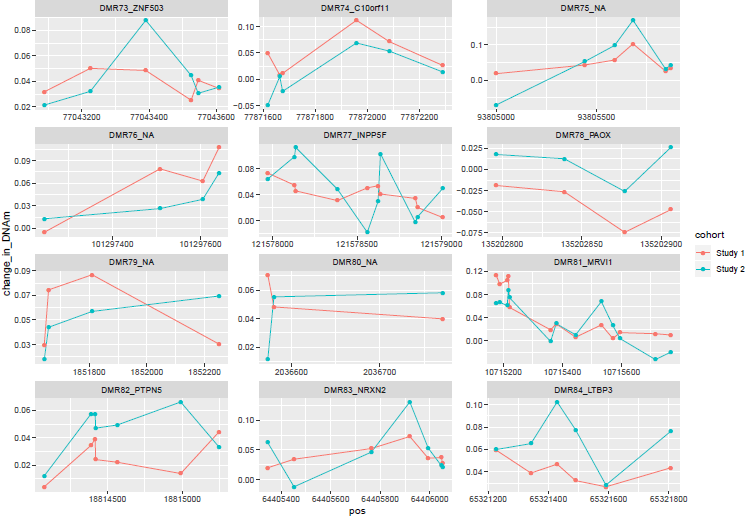


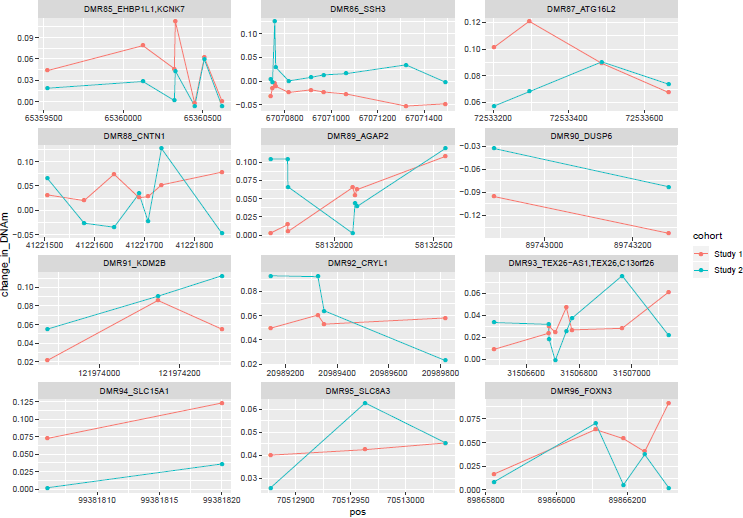


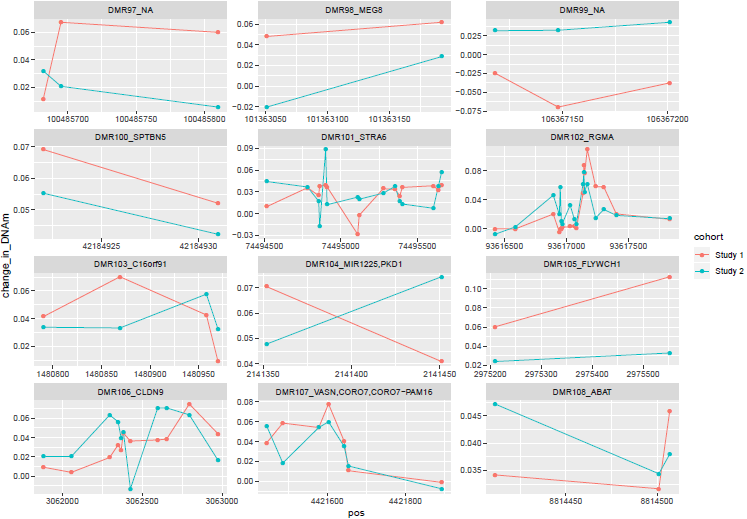


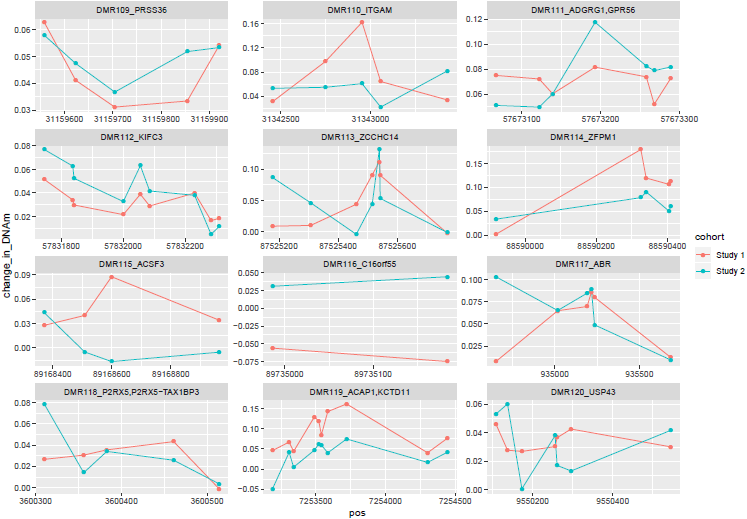


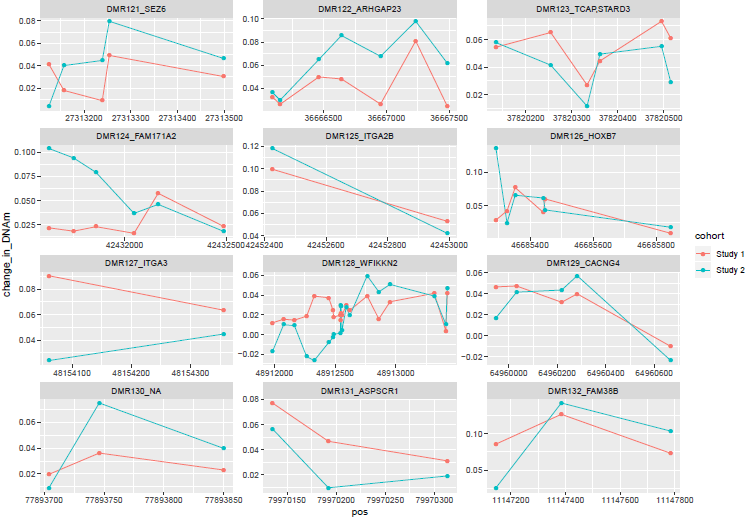


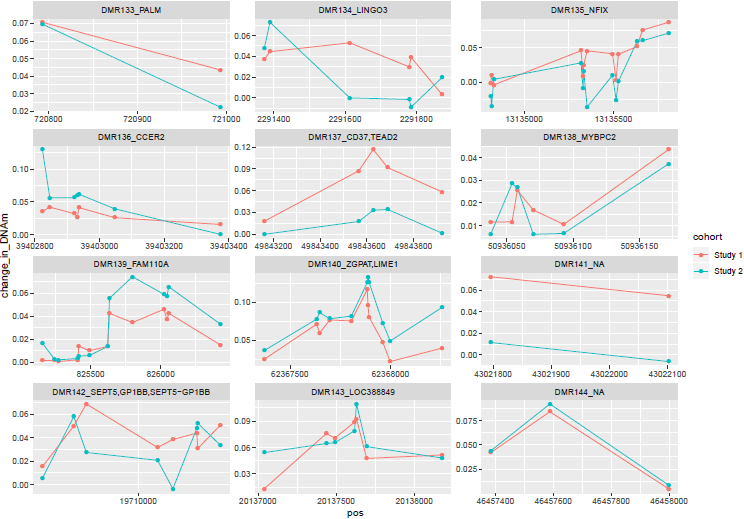


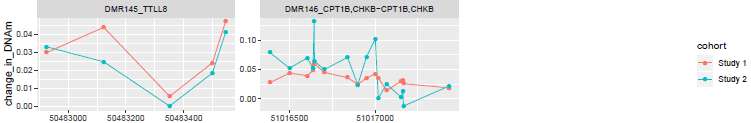


# **Supplementary Figure 2 – Differential methylation levels by individual CpG sites at all DMRs**. Differential methylation is shown for all DMRs for study 1 (B (late) sample – A (early sample) and for study 2 (adult CCS – adult control). Differences shown are differences in beta value and chromosomal location is shown on the X-axis.

# **Supplementary Figure 3**


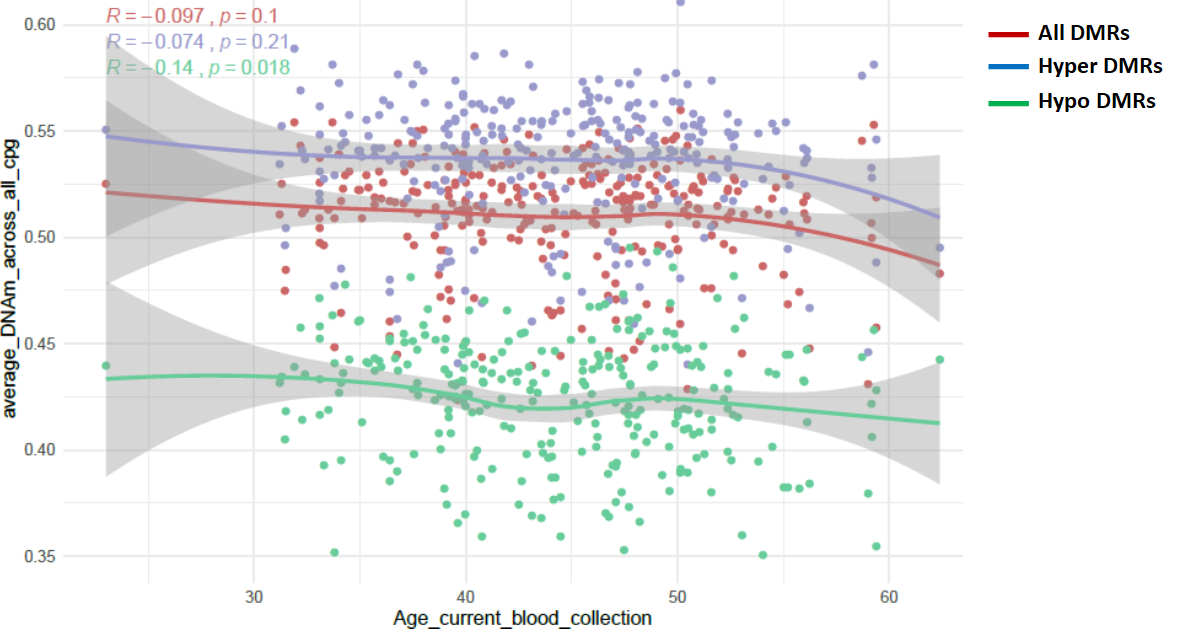


# **Supplementary Figure 3 – DMRs exhibit slight, non-significant, reduction in methylation across ageing in the control population**. Average methylation (beta value) is plotted for all control samples and shown versus age at DNA sampling. Red; average of all DMRs, Blue; average of DMRs hypermethylated in adult CCS, Green; average of DMRs hypomethylated in adult CCS

# Supplementary Tables

**Supplementary Table 1 – Chemotherapeutic agents used in the treatment of patients from Study 1**

| Diagnosis | Agents used for therapy |
| --- | --- |
| ALL | L-Aparaginase, cyclophosphamide, cytarabine, daunorubicin, dexamethasone/prednislone, Etoposide, 6-mercaptopurine, methotrexate, 6-thioguanine, Vincristine |
| AML | AraC, asparaginase, cyclophsphomide, daunorubicin, doxorubicin, etoposide, hydrocortisone |
| NHL | AraC, asparaginase, daunorubicin, etoposide, 6-mercaptopurine, methotrexate, radiotherapy, steroid, 6-thioguanine, vincristine |
| Neuroblastoma | cisplatin, cyclophosphamide, etoposide, melphalan, vincristine |
| Osteosarcoma | cisplatin, carboplatin, doxorubicin, vincristine, melphalan; |
| Wilms' tumour | ActinomycinD, doxorubicin, vincristine |

| **Supplementary Table 2 - Cell type distribution in study 1 and study 2 samples.** | | | | | | |
| --- | --- | --- | --- | --- | --- | --- |
|  |  |  |  |  |  |  |
| **Study 1** | CD8T | CD4T | NK | B-cell | Monocytes | Granulocytes |
| A sample | 0.09 | 0.15 | 0.04 | 0.11 | 0.18 | 0.48 |
| B sample | 0.10 | 0.13 | 0.04 | 0.07 | 0.21 | 0.51 |
| p-Value^a^ | 0.71 | 0.38 | 0.54 | 0.03 | 0.31 | 0.54 |
|  |  |  |  |  |  |  |
| **Study 2** | CD8T | CD4T | NK | B-cell | Monocytes | Granulocytes |
| Adult CCS | 0.08 | 0.12 | 0.06 | 0.06 | 0.08 | 0.63 |
| Control | 0.08 | 0.14 | 0.07 | 0.05 | 0.08 | 0.59 |
| p-Value^a^ | 0.74 | 0.01 | 0.13 | 0.28 | 0.98 | 0.08 |
| Cell type distribution was calculated from genome-wide DNA methylation data, using the Houseman | | | | | | |
| method (see methods for details). | | |  |  |  |  |
| ^a^p-value based on Student T-test (uncorrected p-value) | | | |  |  |  |

Supplementary Table 3 Summary of the DMRs and average changes in DNA methylation across the identified DMRs in child patients (study 1) and adult CCS (study 2, before and after criteria)

| DMR | Nearest gene | Chromosomal location | no.cpgs  DMR | Average beta early vs late remission | Average beta in adult CCS vs controls | Proportion of the DNA methylation change retained (%) | Retained after criteria (n=107) | Average beta  after criteria |
| --- | --- | --- | --- | --- | --- | --- | --- | --- |
| DMR1 | ACAP3 | chr1:1228990-1229589 | 6 | 0.034 | 0.028 | 81.6 | TRUE | 0.023 |
| DMR2 | MIB2 | chr1:1564422-1565027 | 7 | 0.057 | 0.089 | 157.5 | TRUE | 0.078 |
| DMR3 | - | chr1:2885085-2885244 | 4 | 0.038 | 0.052 | 136.8 | TRUE | 0.047 |
| DMR4 | FLJ42875,PRDM16 | chr1:2983926-2984830 | 7 | 0.023 | 0.051 | 223.2 | TRUE | 0.069 |
| DMR5 | PRDM16 | chr1:2987645-2987961 | 3 | 0.032 | 0.043 | 135.1 | TRUE | 0.062 |
| DMR6 | PRDM16 | chr1:2989085-2989307 | 2 | 0.085 | 0.071 | 83.4 | TRUE | 0.065 |
| DMR7 | PRDM16 | chr1:3123695-3123854 | 2 | 0.055 | 0.053 | 96.2 | TRUE | 0.05 |
| DMR8 | PRDM16 | chr1:3239139-3240386 | 5 | 0.082 | 0.062 | 75.7 | TRUE | 0.072 |
| DMR9 | PRDM16 | chr1:3269252-3269478 | 3 | 0.066 | 0.032 | 48.4 | FALSE | - |
| DMR10 | TP73 | chr1:3634811-3635210 | 5 | 0.031 | 0.049 | 159.5 | TRUE | 0.05 |
| DMR11 | NPPA | chr1:11907986-11908659 | 7 | 0.038 | 0 | 0.8 | FALSE | - |
| DMR12 | LYPLA2 | chr1:24120017-24120115 | 3 | 0.038 | 0.053 | 140 | TRUE | 0.051 |
| DMR13 | MAP3K6 | chr1:27683345-27683501 | 4 | 0.041 | 0.051 | 125.1 | TRUE | 0.045 |
| DMR14 | TFAP2E | chr1:36038468-36039356 | 9 | 0.026 | 0.047 | 179 | TRUE | 0.063 |
| DMR15 | DNALI1 | chr1:38021830-38022657 | 11 | 0.021 | 0.026 | 124.5 | TRUE | 0.038 |
| DMR16 | HIVEP3 | chr1:42384002-42385160 | 11 | 0.058 | 0.013 | 21.8 | FALSE | - |
| DMR17 | TNR | chr1:175474437-175474533 | 3 | 0.06 | 0.044 | 72.6 | TRUE | 0.044 |
| DMR18 | NAV1 | chr1:201617346-201619900 | 13 | 0.056 | 0.047 | 84.7 | TRUE | 0.064 |
| DMR19 | GALNT2 | chr1:230414987-230416101 | 9 | 0.049 | 0.021 | 42.9 | FALSE | - |
| DMR20 | UCN | chr2:27530829-27531597 | 11 | 0.023 | 0.023 | 98 | TRUE | 0.025 |
| DMR21 | KRTCAP3,NRBP1 | chr2:27664658-27666036 | 12 | 0.061 | 0.046 | 75.3 | TRUE | 0.07 |
| DMR22 | MEIS1 | chr2:66735693-66736054 | 3 | 0.039 | 0.04 | 103.3 | TRUE | 0.041 |
| DMR23 | IL1RL2 | chr2:102803290-102803787 | 7 | 0.025 | 0.02 | 80.4 | TRUE | 0.03 |
| DMR24 | - | chr2:121498194-121499710 | 7 | 0.044 | 0.03 | 68.2 | TRUE | 0.036 |
| DMR25 | TNS1 | chr2:218769234-218770270 | 6 | 0.05 | 0.032 | 65 | TRUE | 0.05 |
| DMR26 | - | chr2:218843194-218844202 | 8 | 0.034 | 0.052 | 153.7 | TRUE | 0.046 |
| DMR27 | WNT6 | chr2:219737392-219738732 | 6 | 0.072 | 0.077 | 107.6 | TRUE | 0.075 |
| DMR28 | ANKMY1 | chr2:241458974-241459502 | 3 | 0.041 | 0.018 | 44.4 | FALSE | - |
| DMR29 | DNAH1 | chr3:52351599-52351963 | 4 | 0.03 | 0.045 | 152 | TRUE | 0.05 |
| DMR30 | WDR52 | chr3:113160071-113160183 | 2 | 0.032 | -0.008 | -25.6 | FALSE | - |
| DMR31 | - | chr4:1771149-1772151 | 3 | 0.043 | 0.011 | 26 | FALSE | - |
| DMR32 | LIMCH1 | chr4:41614123-41614842 | 8 | 0.03 | 0.001 | 2.1 | FALSE | - |
| DMR33 | PRDM8 | chr4:81111177-81111527 | 5 | 0.04 | 0.011 | 27.6 | FALSE | - |
| DMR34 | PRDM8 | chr4:81117647-81119473 | 14 | 0.124 | 0.092 | 74.1 | TRUE | 0.095 |
| DMR35 | - | chr4:81128029-81128972 | 6 | 0.041 | 0.051 | 123.1 | TRUE | 0.057 |
| DMR36 | - | chr4:183728026-183729343 | 7 | 0.041 | 0.009 | 22 | FALSE | - |
| DMR37 | CMYA5 | chr5:78985425-78986160 | 11 | 0.039 | 0.043 | 109.3 | TRUE | 0.039 |
| DMR38 | SLC25A2 | chr5:140683196-140683946 | 9 | 0.029 | 0.048 | 168.4 | TRUE | 0.048 |
| DMR39 | - | chr5:163723233-163724070 | 11 | 0.045 | 0.016 | 35.8 | FALSE | - |
| DMR40 | NEURL1B | chr5:172110211-172110579 | 4 | 0.043 | 0.047 | 109 | TRUE | 0.055 |
| DMR41 | PFN3 | chr5:176827082-176828378 | 8 | 0.029 | 0.027 | 95.5 | TRUE | 0.037 |
| DMR42 | GCNT2 | chr6:10555682-10556523 | 9 | 0.022 | 0.011 | 51 | FALSE | - |
| DMR43 | FAM65B | chr6:24910562-24911615 | 17 | 0.039 | 0.017 | 44.2 | FALSE | - |
| DMR44 | HIST1H3E | chr6:26224668-26225767 | 10 | 0.03 | 0.002 | 5.1 | FALSE | - |
| DMR45 | - | chr6:30094960-30095802 | 25 | 0.02 | 0.047 | 229.1 | TRUE | 0.054 |
| DMR46 | KIAA1949,NRM | chr6:30656499-30656667 | 5 | 0.036 | 0.036 | 101.6 | TRUE | 0.038 |
| DMR47 | DDR1 | chr6:30852354-30854551 | 26 | 0.033 | 0.042 | 125.8 | TRUE | 0.048 |
| DMR48 | DDR1 | chr6:30859697-30860960 | 18 | 0.026 | 0.022 | 85.4 | TRUE | 0.032 |
| DMR49 | VARS2,GTF2H4 | chr6:30881152-30881484 | 4 | 0.022 | 0.007 | 31.5 | FALSE | - |
| DMR50 | VARS | chr6:31760047-31761076 | 14 | 0.04 | 0.034 | 86.5 | TRUE | 0.051 |
| DMR51 | HLA-DPB1 | chr6:33047944-33048879 | 15 | 0.019 | 0.05 | 258.4 | TRUE | 0.043 |
| DMR52 | HLA-DPB2 | chr6:33084479-33085063 | 15 | 0.019 | 0.043 | 222.2 | TRUE | 0.04 |
| DMR53 | COL11A2 | chr6:33141040-33141305 | 4 | 0.039 | 0.047 | 119 | TRUE | 0.035 |
| DMR54 | DDO | chr6:110736422-110737053 | 6 | -0.054 | -0.008 | 15.2 | FALSE | - |
| DMR55 | MAD1L1 | chr7:1882997-1883760 | 7 | 0.047 | 0.037 | 78.8 | TRUE | 0.064 |
| DMR56 | RADIL | chr7:4859229-4859763 | 5 | 0.034 | 0.02 | 58.3 | TRUE | 0.024 |
| DMR57 | HOXA4 | chr7:27168962-27171528 | 30 | 0.061 | 0.054 | 89.3 | TRUE | 0.064 |
| DMR58 | HOXA5 | chr7:27184264-27184853 | 14 | 0.032 | 0.025 | 79.1 | TRUE | 0.033 |
| DMR59 | TNS3 | chr7:47576580-47577090 | 4 | 0.052 | 0.037 | 71.2 | TRUE | 0.056 |
| DMR60 | SAP25,LRCH4,ZASP | chr7:100171617-100172527 | 6 | 0.023 | 0.048 | 208.6 | TRUE | 0.046 |
| DMR61 | AGAP3 | chr7:150822655-150823386 | 4 | 0.045 | 0.044 | 97.7 | TRUE | 0.044 |
| DMR62 | PIWIL2 | chr8:22132434-22133356 | 14 | 0.022 | 0.021 | 98.2 | TRUE | 0.054 |
| DMR63 | LOC101929237,PEBP4 | chr8:22734960-22735478 | 6 | 0.038 | 0.042 | 109.8 | TRUE | 0.034 |
| DMR64 | TRAPPC9 | chr8:141057285-141057827 | 4 | 0.04 | 0.022 | 55.7 | TRUE | 0.032 |
| DMR65 | KIAA1161 | chr9:34370781-34371380 | 5 | 0.032 | 0.012 | 39.5 | FALSE | - |
| DMR66 | MAMDC2 | chr9:72657974-72658747 | 6 | 0.033 | 0.023 | 69.5 | TRUE | 0.045 |
| DMR67 | TRIM14 | chr9:100850334-100850391 | 3 | 0.126 | 0.048 | 38 | FALSE | - |
| DMR68 | LHX6 | chr9:124990010-124991656 | 18 | 0.036 | 0.028 | 77.2 | TRUE | 0.038 |
| DMR69 | PTGS1 | chr9:125137380-125137594 | 6 | 0.075 | 0.03 | 40 | FALSE | - |
| DMR70 | C10orf47 | chr10:11911721-11912410 | 3 | 0.056 | 0.051 | 90.2 | TRUE | 0.032 |
| DMR71 | ALOX5 | chr10:45922877-45923705 | 5 | 0.031 | 0.017 | 55.3 | TRUE | 0.039 |
| DMR72 | - | chr10:72219713-72219819 | 3 | 0.045 | 0.068 | 151.1 | TRUE | 0.06 |
| DMR73 | ZNF503 | chr10:77043088-77043608 | 6 | 0.037 | 0.042 | 114.7 | TRUE | 0.046 |
| DMR74 | C10orf11 | chr10:77871618-77872288 | 7 | 0.041 | 0.012 | 28.4 | FALSE | 0.07 |
| DMR75 | - | chr10:93804999-93805870 | 6 | 0.045 | 0.054 | 120.3 | TRUE | 0.052 |
| DMR76 | - | chr10:101297245-101297642 | 4 | 0.061 | 0.038 | 62 | TRUE | - |
| DMR77 | INPP5F | chr10:121577971-121579007 | 10 | 0.039 | 0.049 | 127 | TRUE | 0.057 |
| DMR78 | PAOX | chr10:135202796-135202906 | 3 | -0.031 | 0.008 | -24.5 | FALSE | - |
| DMR79 | - | chr11:1851644-1852250 | 4 | 0.054 | 0.047 | 87.7 | TRUE | 0.051 |
| DMR80 | - | chr11:2036573-2036771 | 3 | 0.051 | 0.042 | 82.1 | TRUE | 0.049 |
| DMR81 | MRVI1 | chr11:10715175-10715767 | 13 | 0.044 | 0.034 | 76.3 | TRUE | 0.044 |
| DMR82 | PTPN5 | chr11:18814078-18815245 | 6 | 0.031 | 0.046 | 147.5 | TRUE | 0.042 |
| DMR83 | NRXN2 | chr11:64405346-64406051 | 7 | 0.039 | 0.046 | 118.7 | TRUE | 0.052 |
| DMR84 | LTBP3 | chr11:65321226-65321806 | 6 | 0.04 | 0.068 | 169.2 | TRUE | 0.059 |
| DMR85 | EHBP1L1,KCNK7 | chr11:65359521-65360620 | 7 | 0.047 | 0.02 | 41.8 | FALSE | - |
| DMR86 | SSH3 | chr11:67070738-67071492 | 10 | -0.025 | 0.023 | -90.2 | FALSE | - |
| DMR87 | ATG16L2 | chr11:72533202-72533664 | 4 | 0.09 | 0.072 | 80.5 | TRUE | 0.066 |
| DMR88 | CNTN1 | chr12:41221505-41222241 | 8 | 0.047 | 0.014 | 29.2 | FALSE | 0.051 |
| DMR89 | AGAP2 | chr12:58131681-58133008 | 9 | 0.04 | 0.068 | 171.6 | TRUE | -0.055 |
| DMR90 | DUSP6 | chr12:89742886-89743281 | 2 | -0.115 | -0.058 | 50.3 | TRUE | - |
| DMR91 | KDM2B | chr12:121973871-121974305 | 3 | 0.053 | 0.086 | 163.4 | TRUE | 0.085 |
| DMR92 | CRYL1 | chr13:20989142-20989818 | 4 | 0.053 | 0.068 | 128.1 | TRUE | 0.084 |
| DMR93 | TEX26-AS1,TEX26, C13orf26 | chr13:31506479-31507139 | 9 | 0.028 | 0.03 | 109.9 | TRUE | 0.033 |
| DMR94 | SLC15A1 | chr13:99381806-99381820 | 2 | 0.098 | 0.019 | 19.5 | FALSE | - |
| DMR95 | SLC8A3 | chr14:70512877-70513036 | 3 | 0.042 | 0.045 | 107.4 | TRUE | 0.038 |
| DMR96 | FOXN3 | chr14:89865823-89866317 | 4 | 0.049 | 0.025 | 50.4 | TRUE | 0.028 |
| DMR97 | - | chr14:100485682-100485810 | 3 | 0.046 | 0.019 | 41.6 | FALSE | - |
| DMR98 | MEG8 | chr14:101363051-101363192 | 2 | 0.053 | 0.004 | 7.8 | FALSE | - |
| DMR99 | - | chr14:106367118-106367201 | 3 | -0.045 | 0.036 | -79.5 | FALSE | - |
| DMR100 | SPTBN5 | chr15:42184922-42184931 | 2 | 0.059 | 0.049 | 82.9 | TRUE | 0.049 |
| DMR101 | STRA6 | chr15:74494515-74495657 | 15 | 0.025 | 0.028 | 113.3 | TRUE | 0.046 |
| DMR102 | RGMA | chr15:93616424-93617833 | 18 | 0.026 | 0.029 | 109.2 | TRUE | 0.019 |
| DMR103 | C16orf91 | chr16:1480790-1480970 | 4 | 0.039 | 0.039 | 101.8 | TRUE | 0.046 |
| DMR104 | MIR1225,PKD1 | chr16:2141352-2141451 | 2 | 0.057 | 0.061 | 107.8 | TRUE | 0.039 |
| DMR105 | FLYWCH1 | chr16:2975209-2975552 | 2 | 0.084 | 0.028 | 33.5 | FALSE | 0.044 |
| DMR106 | CLDN9 | chr16:3061880-3062975 | 11 | 0.033 | 0.041 | 124.2 | TRUE | 0.038 |
| DMR107 | VASN,CORO7,CORO7-PAM16 | chr16:4421445-4421892 | 7 | 0.038 | 0.033 | 86 | TRUE | 0.048 |
| DMR108 | ABAT | chr16:8814411-8814755 | 4 | 0.029 | 0.04 | 136.2 | TRUE | 0.041 |
| DMR109 | PRSS36 | chr16:31159558-31159920 | 5 | 0.043 | 0.049 | 113.7 | TRUE | 0.04 |
| DMR110 | ITGAM | chr16:31342453-31343428 | 5 | 0.078 | 0.055 | 70.4 | TRUE | 0.052 |
| DMR111 | ADGRG1,GPR56 | chr16:57673067-57673289 | 6 | 0.069 | 0.074 | 107.8 | TRUE | 0.062 |
| DMR112 | KIFC3 | chr16:57831745-57832309 | 10 | 0.031 | 0.043 | 139.8 | TRUE | 0.043 |
| DMR113 | ZCCHC14 | chr16:87525178-87525769 | 7 | 0.046 | 0.051 | 110.4 | TRUE | 0.048 |
| DMR114 | ZFPM1 | chr16:88589918-88590409 | 5 | 0.097 | 0.063 | 64.4 | TRUE | 0.07 |
| DMR115 | ACSF3 | chr16:89168371-89168963 | 4 | 0.045 | 0.004 | 9.1 | FALSE | - |
| DMR116 | C16orf55 | chr16:89734986-89735184 | 2 | -0.066 | 0.038 | -56.8 | FALSE | - |
| DMR117 | ABR | chr17:934652-935766 | 7 | 0.046 | 0.067 | 144 | TRUE | 0.067 |
| DMR118 | P2RX5,P2RX5-TAX1BP3 | chr17:3600310-3600514 | 5 | 0.026 | 0.031 | 119 | TRUE | 0.036 |
| DMR119 | ACAP1,KCTD11 | chr17:7253189-7254443 | 10 | 0.089 | 0.034 | 38.3 | FALSE | - |
| DMR120 | USP43 | chr17:9550108-9550545 | 7 | 0.035 | 0.032 | 92.4 | TRUE | 0.025 |
| DMR121 | SEZ6 | chr17:27313125-27313499 | 5 | 0.026 | 0.043 | 167.6 | TRUE | 0.049 |
| DMR122 | ARHGAP23 | chr17:36666093-36667484 | 7 | 0.04 | 0.064 | 157.6 | TRUE | 0.062 |
| DMR123 | TCAP,STARD3 | chr17:37820135-37820515 | 6 | 0.053 | 0.041 | 77 | TRUE | 0.041 |
| DMR124 | FAM171A2 | chr17:42431627-42432488 | 6 | 0.026 | 0.063 | 238.5 | TRUE | 0.063 |
| DMR125 | ITGA2B | chr17:42452426-42452994 | 2 | 0.075 | 0.08 | 107.1 | TRUE | 0.074 |
| DMR126 | HOXB7 | chr17:46685292-46685846 | 6 | 0.041 | 0.058 | 142.7 | TRUE | 0.057 |
| DMR127 | ITGA3 | chr17:48154061-48154356 | 2 | 0.074 | 0.034 | 46.6 | FALSE | - |
| DMR128 | WFIKKN2 | chr17:48911983-48913424 | 20 | 0.023 | 0.015 | 65.4 | TRUE | 0.027 |
| DMR129 | CACNG4 | chr17:64959947-64960668 | 5 | 0.031 | 0.027 | 88.4 | TRUE | 0.037 |
| DMR130 | - | chr17:77893704-77893850 | 3 | 0.025 | 0.041 | 165.5 | TRUE | 0.043 |
| DMR131 | ASPSCR1 | chr17:79970135-79970313 | 3 | 0.049 | 0.029 | 57.8 | FALSE | - |
| DMR132 | FAM38B | chr18:11147146-11147785 | 3 | 0.092 | 0.09 | 98.1 | TRUE | 0.081 |
| DMR133 | PALM | chr19:720793-720993 | 2 | 0.056 | 0.046 | 82.2 | FALSE | - |
| DMR134 | LINGO3 | chr19:2291373-2291872 | 6 | 0.033 | 0.022 | 65.7 | TRUE | 0.063 |
| DMR135 | NFIX | chr19:13134813-13135808 | 13 | 0.032 | 0.01 | 30.7 | FALSE | - |
| DMR136 | CCER2 | chr19:39402823-39403373 | 7 | 0.029 | 0.058 | 201.8 | TRUE | 0.059 |
| DMR137 | CD37,TEAD2 | chr19:49843160-49843922 | 5 | 0.073 | 0.017 | 23.2 | FALSE | - |
| DMR138 | MYBPC2 | chr19:50936038-50936172 | 6 | 0.02 | 0.019 | 91.7 | TRUE | 0.027 |
| DMR139 | FAM110A | chr20:825155-826424 | 13 | 0.018 | 0.03 | 164 | TRUE | 0.052 |
| DMR140 | ZGPAT,LIME1 | chr20:62367372-62368256 | 12 | 0.06 | 0.087 | 145.3 | TRUE | 0.067 |
| DMR141 | - | chr21:43021796-43022102 | 2 | 0.062 | 0.003 | 4.2 | FALSE | - |
| DMR142 | SEPT5,GP1BB,SEPT5-GP1BB | chr22:19709548-19710386 | 8 | 0.039 | 0.03 | 78.3 | TRUE | 0.044 |
| DMR143 | LOC388849 | chr22:20137040-20138177 | 7 | 0.062 | 0.069 | 111.9 | TRUE | 0.064 |
| DMR144 | - | chr22:46457384-46457998 | 3 | 0.043 | 0.048 | 112 | TRUE | 0.048 |
| DMR145 | TTLL8 | chr22:50482925-50483545 | 5 | 0.029 | 0.024 | 81.6 | FALSE | - |
| DMR146 | CPT1B,CHKB-CPT1B,CHKB | chr22:51016386-51017432 | 16 | 0.035 | 0.048 | 137.1 | TRUE | 0.061 |

DMRs were defined as being retained in the adult survivors if they were statistically significantly different from the control population, exhibited the same direction of change as seen following exposure to treatment, and had an absolute difference in methylation of at least 50% of the change identified in the post-treatment samples.

Supplementary Table 4 Differences in DMR methylation between haematological and solid malignancies in childhood cancer patients (study 1).

| DMR | Gene | Difference | Mean Haem | Mean Solid | p.value | conf.low | conf.high | FDR |
| --- | --- | --- | --- | --- | --- | --- | --- | --- |
| Average | NA | -0.020 | 0.043 | 0.062 | 0.176 | -0.050 | 0.011 | 0.446 |
| DMR1 | ACAP3 | -0.012 | 0.031 | 0.044 | 0.582 | -0.062 | 0.038 | 0.759 |
| DMR2 | MIB2 | -0.022 | 0.053 | 0.075 | 0.311 | -0.068 | 0.024 | 0.586 |
| DMR3 | NA | 0.006 | 0.042 | 0.036 | 0.742 | -0.031 | 0.042 | 0.820 |
| DMR4 | FLJ42875,PRDM16 | -0.003 | 0.018 | 0.021 | 0.620 | -0.017 | 0.010 | 0.785 |
| DMR5 | PRDM16 | -0.007 | 0.033 | 0.040 | 0.407 | -0.026 | 0.011 | 0.657 |
| DMR6 | PRDM16 | -0.017 | 0.083 | 0.099 | 0.442 | -0.060 | 0.027 | 0.664 |
| DMR7 | PRDM16 | -0.051 | 0.049 | 0.099 | 0.020 | -0.092 | -0.010 | 0.138 |
| DMR8 | PRDM16 | -0.068 | 0.070 | 0.138 | 0.027 | -0.128 | -0.009 | 0.138 |
| DMR9 | PRDM16 | -0.056 | 0.055 | 0.110 | 0.038 | -0.107 | -0.004 | 0.176 |
| DMR10 | TP73 | -0.004 | 0.031 | 0.036 | 0.681 | -0.028 | 0.019 | 0.801 |
| DMR11 | NPPA | -0.041 | 0.031 | 0.071 | 0.017 | -0.073 | -0.008 | 0.138 |
| DMR12 | LYPLA2 | -0.009 | 0.037 | 0.046 | 0.589 | -0.044 | 0.026 | 0.760 |
| DMR13 | MAP3K6 | -0.032 | 0.033 | 0.065 | 0.255 | -0.090 | 0.027 | 0.533 |
| DMR14 | TFAP2E | -0.039 | 0.019 | 0.058 | 0.013 | -0.068 | -0.010 | 0.138 |
| DMR15 | DNALI1 | -0.013 | 0.019 | 0.032 | 0.182 | -0.032 | 0.007 | 0.446 |
| DMR16 | HIVEP3 | -0.107 | 0.034 | 0.141 | 0.017 | -0.189 | -0.025 | 0.138 |
| DMR17 | TNR | -0.063 | 0.048 | 0.112 | 0.044 | -0.124 | -0.002 | 0.180 |
| DMR18 | NAV1 | -0.017 | 0.052 | 0.070 | 0.340 | -0.057 | 0.022 | 0.625 |
| DMR19 | GALNT2 | -0.017 | 0.046 | 0.063 | 0.454 | -0.066 | 0.033 | 0.668 |
| DMR20 | UCN | -0.005 | 0.023 | 0.028 | 0.724 | -0.038 | 0.027 | 0.819 |
| DMR21 | KRTCAP3,NRBP1 | -0.038 | 0.056 | 0.094 | 0.178 | -0.096 | 0.021 | 0.446 |
| DMR22 | MEIS1 | -0.046 | 0.030 | 0.076 | 0.020 | -0.083 | -0.008 | 0.138 |
| DMR23 | IL1RL2 | -0.018 | 0.022 | 0.040 | 0.284 | -0.054 | 0.018 | 0.564 |
| DMR24 | NA | -0.023 | 0.040 | 0.063 | 0.211 | -0.062 | 0.016 | 0.464 |
| DMR25 | TNS1 | -0.035 | 0.043 | 0.079 | 0.061 | -0.073 | 0.002 | 0.209 |
| DMR26 | NA | -0.011 | 0.031 | 0.042 | 0.352 | -0.036 | 0.014 | 0.630 |
| DMR27 | WNT6 | 0.040 | 0.076 | 0.036 | 0.006 | 0.013 | 0.068 | 0.138 |
| DMR28 | ANKMY1 | -0.055 | 0.031 | 0.086 | 0.024 | -0.101 | -0.009 | 0.138 |
| DMR29 | DNAH1 | -0.010 | 0.030 | 0.040 | 0.411 | -0.035 | 0.016 | 0.657 |
| DMR30 | WDR52 | -0.026 | 0.028 | 0.054 | 0.204 | -0.070 | 0.017 | 0.464 |
| DMR31 | NA | -0.040 | 0.038 | 0.078 | 0.047 | -0.080 | -0.001 | 0.180 |
| DMR32 | LIMCH1 | 0.006 | 0.030 | 0.024 | 0.423 | -0.009 | 0.021 | 0.657 |
| DMR33 | PRDM8 | -0.049 | 0.030 | 0.079 | 0.047 | -0.098 | -0.001 | 0.180 |
| DMR34 | PRDM8 | -0.094 | 0.105 | 0.199 | 0.044 | -0.185 | -0.003 | 0.180 |
| DMR35 | NA | -0.024 | 0.035 | 0.059 | 0.295 | -0.074 | 0.026 | 0.571 |
| DMR36 | NA | -0.029 | 0.037 | 0.066 | 0.126 | -0.067 | 0.010 | 0.348 |
| DMR37 | CMYA5 | 0.013 | 0.045 | 0.032 | 0.531 | -0.032 | 0.059 | 0.721 |
| DMR38 | SLC25A2 | -0.014 | 0.027 | 0.042 | 0.351 | -0.047 | 0.018 | 0.630 |
| DMR39 | NA | 0.009 | 0.046 | 0.038 | 0.375 | -0.011 | 0.028 | 0.648 |
| DMR40 | NEURL1B | -0.009 | 0.043 | 0.051 | 0.659 | -0.052 | 0.034 | 0.797 |
| DMR41 | PFN3 | -0.003 | 0.029 | 0.032 | 0.795 | -0.025 | 0.020 | 0.853 |
| DMR42 | GCNT2 | 0.004 | 0.024 | 0.020 | 0.720 | -0.020 | 0.028 | 0.819 |
| DMR43 | FAM65B | -0.051 | 0.029 | 0.080 | 0.090 | -0.111 | 0.010 | 0.270 |
| DMR44 | HIST1H3E | -0.011 | 0.037 | 0.048 | 0.435 | -0.041 | 0.019 | 0.664 |
| DMR45 | NA | 0.016 | 0.027 | 0.011 | 0.208 | -0.010 | 0.042 | 0.464 |
| DMR46 | KIAA1949,NRM | 0.002 | 0.033 | 0.031 | 0.908 | -0.038 | 0.042 | 0.928 |
| DMR47 | DDR1 | 0.008 | 0.037 | 0.029 | 0.488 | -0.017 | 0.034 | 0.704 |
| DMR48 | DDR1 | -0.011 | 0.025 | 0.036 | 0.424 | -0.041 | 0.018 | 0.657 |
| DMR49 | VARS2,GTF2H4 | -0.043 | 0.015 | 0.058 | 0.003 | -0.069 | -0.017 | 0.135 |
| DMR50 | VARS | -0.070 | 0.027 | 0.096 | 0.008 | -0.117 | -0.023 | 0.138 |
| DMR51 | HLA-DPB1 | -0.012 | 0.018 | 0.030 | 0.416 | -0.043 | 0.020 | 0.657 |
| DMR52 | HLA-DPB2 | 0.014 | 0.021 | 0.008 | 0.026 | 0.002 | 0.026 | 0.138 |
| DMR53 | COL11A2 | 0.043 | 0.047 | 0.004 | 0.015 | 0.010 | 0.077 | 0.138 |
| DMR54 | DDO | -0.010 | -0.054 | -0.044 | 0.688 | -0.062 | 0.042 | 0.803 |
| DMR55 | MAD1L1 | -0.027 | 0.044 | 0.071 | 0.363 | -0.091 | 0.036 | 0.635 |
| DMR56 | RADIL | -0.022 | 0.029 | 0.051 | 0.244 | -0.062 | 0.018 | 0.525 |
| DMR57 | HOXA4 | 0.000 | 0.064 | 0.063 | 0.984 | -0.046 | 0.047 | 0.991 |
| DMR58 | HOXA5 | -0.078 | 0.016 | 0.095 | 0.009 | -0.132 | -0.025 | 0.138 |
| DMR59 | TNS3 | -0.044 | 0.038 | 0.082 | 0.068 | -0.092 | 0.004 | 0.216 |
| DMR60 | SAP25,LRCH4,ZASP | -0.038 | 0.020 | 0.058 | 0.046 | -0.075 | -0.001 | 0.180 |
| DMR61 | AGAP3 | -0.010 | 0.044 | 0.054 | 0.542 | -0.046 | 0.025 | 0.724 |
| DMR62 | PIWIL2 | -0.010 | 0.022 | 0.032 | 0.505 | -0.044 | 0.024 | 0.707 |
| DMR63 | LOC101929237,PEBP4 | 0.020 | 0.044 | 0.024 | 0.392 | -0.031 | 0.071 | 0.657 |
| DMR64 | TRAPPC9 | -0.017 | 0.038 | 0.054 | 0.455 | -0.065 | 0.032 | 0.668 |
| DMR65 | KIAA1161 | -0.009 | 0.032 | 0.042 | 0.662 | -0.058 | 0.039 | 0.797 |
| DMR66 | MAMDC2 | 0.008 | 0.034 | 0.026 | 0.583 | -0.025 | 0.042 | 0.759 |
| DMR67 | TRIM14 | -0.111 | 0.116 | 0.227 | 0.066 | -0.231 | 0.009 | 0.216 |
| DMR68 | LHX6 | 0.006 | 0.039 | 0.033 | 0.721 | -0.029 | 0.040 | 0.819 |
| DMR69 | PTGS1 | -0.051 | 0.065 | 0.116 | 0.070 | -0.107 | 0.005 | 0.220 |
| DMR70 | C10orf47 | -0.007 | 0.055 | 0.063 | 0.788 | -0.070 | 0.055 | 0.852 |
| DMR71 | ALOX5 | 0.000 | 0.033 | 0.033 | 0.991 | -0.024 | 0.024 | 0.991 |
| DMR72 | NA | 0.025 | 0.051 | 0.026 | 0.112 | -0.007 | 0.056 | 0.323 |
| DMR73 | ZNF503 | -0.044 | 0.029 | 0.073 | 0.005 | -0.073 | -0.016 | 0.135 |
| DMR74 | C10orf11 | 0.028 | 0.050 | 0.022 | 0.015 | 0.006 | 0.050 | 0.138 |
| DMR75 | NA | -0.027 | 0.041 | 0.067 | 0.264 | -0.078 | 0.025 | 0.539 |
| DMR76 | NA | -0.003 | 0.060 | 0.064 | 0.872 | -0.044 | 0.038 | 0.915 |
| DMR77 | INPP5F | -0.010 | 0.039 | 0.049 | 0.656 | -0.061 | 0.041 | 0.797 |
| DMR78 | PAOX | 0.012 | -0.039 | -0.051 | 0.513 | -0.026 | 0.051 | 0.711 |
| DMR79 | NA | -0.029 | 0.049 | 0.078 | 0.165 | -0.072 | 0.014 | 0.433 |
| DMR80 | NA | 0.017 | 0.057 | 0.039 | 0.402 | -0.027 | 0.062 | 0.657 |
| DMR81 | MRVI1 | -0.041 | 0.038 | 0.079 | 0.051 | -0.082 | 0.000 | 0.182 |
| DMR82 | PTPN5 | 0.002 | 0.027 | 0.025 | 0.844 | -0.021 | 0.025 | 0.893 |
| DMR83 | NRXN2 | -0.003 | 0.040 | 0.042 | 0.884 | -0.039 | 0.034 | 0.915 |
| DMR84 | LTBP3 | -0.016 | 0.038 | 0.054 | 0.474 | -0.065 | 0.033 | 0.690 |
| DMR85 | EHBP1L1,KCNK7 | -0.068 | 0.034 | 0.101 | 0.073 | -0.143 | 0.008 | 0.224 |
| DMR86 | SSH3 | -0.005 | -0.027 | -0.022 | 0.750 | -0.035 | 0.026 | 0.822 |
| DMR87 | ATG16L2 | -0.042 | 0.086 | 0.127 | 0.327 | -0.135 | 0.051 | 0.608 |
| DMR88 | CNTN1 | 0.007 | 0.046 | 0.039 | 0.739 | -0.041 | 0.056 | 0.820 |
| DMR89 | AGAP2 | -0.037 | 0.032 | 0.069 | 0.144 | -0.089 | 0.016 | 0.391 |
| DMR90 | DUSP6 | -0.018 | -0.123 | -0.106 | 0.603 | -0.088 | 0.052 | 0.770 |
| DMR91 | KDM2B | -0.028 | 0.048 | 0.076 | 0.186 | -0.072 | 0.016 | 0.448 |
| DMR92 | CRYL1 | -0.028 | 0.049 | 0.077 | 0.290 | -0.083 | 0.028 | 0.568 |
| DMR93 | TEX26-AS1,TEX26,C13orf26 | 0.016 | 0.033 | 0.017 | 0.257 | -0.014 | 0.047 | 0.533 |
| DMR94 | SLC15A1 | -0.052 | 0.086 | 0.138 | 0.197 | -0.136 | 0.032 | 0.464 |
| DMR95 | SLC8A3 | -0.008 | 0.041 | 0.049 | 0.670 | -0.051 | 0.034 | 0.801 |
| DMR96 | FOXN3 | 0.003 | 0.054 | 0.051 | 0.909 | -0.063 | 0.070 | 0.928 |
| DMR97 | NA | -0.032 | 0.039 | 0.071 | 0.246 | -0.091 | 0.027 | 0.525 |
| DMR98 | MEG8 | -0.014 | 0.052 | 0.065 | 0.499 | -0.057 | 0.030 | 0.706 |
| DMR99 | NA | -0.080 | -0.061 | 0.019 | 0.013 | -0.139 | -0.022 | 0.138 |
| DMR100 | SPTBN5 | -0.011 | 0.058 | 0.069 | 0.638 | -0.059 | 0.038 | 0.791 |
| DMR101 | STRA6 | 0.027 | 0.032 | 0.005 | 0.031 | 0.003 | 0.051 | 0.151 |
| DMR102 | RGMA | -0.038 | 0.019 | 0.057 | 0.009 | -0.064 | -0.012 | 0.138 |
| DMR103 | C16orf91 | -0.024 | 0.036 | 0.059 | 0.206 | -0.063 | 0.015 | 0.464 |
| DMR104 | MIR1225,PKD1 | -0.015 | 0.052 | 0.067 | 0.681 | -0.096 | 0.066 | 0.801 |
| DMR105 | FLYWCH1 | -0.079 | 0.069 | 0.149 | 0.017 | -0.140 | -0.018 | 0.138 |
| DMR106 | CLDN9 | -0.011 | 0.031 | 0.042 | 0.493 | -0.046 | 0.023 | 0.704 |
| DMR107 | VASN,CORO7,CORO7-PAM16 | -0.052 | 0.029 | 0.081 | 0.022 | -0.095 | -0.010 | 0.138 |
| DMR108 | ABAT | -0.009 | 0.030 | 0.039 | 0.441 | -0.034 | 0.016 | 0.664 |
| DMR109 | PRSS36 | -0.003 | 0.044 | 0.047 | 0.882 | -0.050 | 0.043 | 0.915 |
| DMR110 | ITGAM | 0.047 | 0.088 | 0.041 | 0.043 | 0.002 | 0.092 | 0.180 |
| DMR111 | ADGRG1,GPR56 | -0.133 | 0.040 | 0.174 | 0.005 | -0.216 | -0.050 | 0.135 |
| DMR112 | KIFC3 | -0.015 | 0.029 | 0.044 | 0.393 | -0.052 | 0.022 | 0.657 |
| DMR113 | ZCCHC14 | -0.070 | 0.035 | 0.106 | 0.023 | -0.128 | -0.012 | 0.138 |
| DMR114 | ZFPM1 | -0.101 | 0.082 | 0.183 | 0.049 | -0.202 | 0.000 | 0.181 |
| DMR115 | ACSF3 | -0.044 | 0.038 | 0.082 | 0.026 | -0.081 | -0.006 | 0.138 |
| DMR116 | C16orf55 | -0.064 | -0.079 | -0.016 | 2.00E-04 | -0.094 | -0.033 | **0.029** |
| DMR117 | ABR | -0.044 | 0.039 | 0.082 | 0.064 | -0.090 | 0.003 | 0.213 |
| DMR118 | P2RX5,P2RX5-TAX1BP3 | -0.005 | 0.026 | 0.031 | 0.733 | -0.039 | 0.029 | 0.820 |
| DMR119 | ACAP1,KCTD11 | -0.150 | 0.059 | 0.208 | 0.004 | -0.240 | -0.060 | 0.135 |
| DMR120 | USP43 | 0.012 | 0.037 | 0.025 | 0.410 | -0.019 | 0.042 | 0.657 |
| DMR121 | SEZ6 | -0.015 | 0.026 | 0.041 | 0.357 | -0.051 | 0.021 | 0.632 |
| DMR122 | ARHGAP23 | -0.001 | 0.041 | 0.042 | 0.946 | -0.026 | 0.024 | 0.959 |
| DMR123 | TCAP,STARD3 | -0.040 | 0.046 | 0.086 | 0.181 | -0.101 | 0.021 | 0.446 |
| DMR124 | FAM171A2 | -0.016 | 0.023 | 0.039 | 0.274 | -0.048 | 0.016 | 0.551 |
| DMR125 | ITGA2B | -0.033 | 0.069 | 0.102 | 0.205 | -0.089 | 0.022 | 0.464 |
| DMR126 | HOXB7 | 0.027 | 0.049 | 0.021 | 0.112 | -0.007 | 0.062 | 0.323 |
| DMR127 | ITGA3 | 0.047 | 0.087 | 0.040 | 0.059 | -0.002 | 0.096 | 0.208 |
| DMR128 | WFIKKN2 | -0.011 | 0.022 | 0.033 | 0.414 | -0.040 | 0.018 | 0.657 |
| DMR129 | CACNG4 | 0.003 | 0.032 | 0.029 | 0.779 | -0.021 | 0.027 | 0.848 |
| DMR130 | NA | 0.005 | 0.027 | 0.022 | 0.640 | -0.018 | 0.029 | 0.791 |
| DMR131 | ASPSCR1 | -0.044 | 0.042 | 0.086 | 0.124 | -0.104 | 0.016 | 0.348 |
| DMR132 | FAM38B | 0.016 | 0.098 | 0.083 | 0.569 | -0.044 | 0.075 | 0.753 |
| DMR133 | PALM | -0.061 | 0.044 | 0.105 | 0.012 | -0.106 | -0.017 | 0.138 |
| DMR134 | LINGO3 | -0.032 | 0.028 | 0.059 | 0.148 | -0.078 | 0.014 | 0.395 |
| DMR135 | NFIX | -0.005 | 0.032 | 0.037 | 0.639 | -0.029 | 0.019 | 0.791 |
| DMR136 | CCER2 | 0.010 | 0.034 | 0.023 | 0.534 | -0.026 | 0.047 | 0.721 |
| DMR137 | CD37,TEAD2 | -0.061 | 0.061 | 0.122 | 0.048 | -0.121 | -0.001 | 0.180 |
| DMR138 | MYBPC2 | -0.019 | 0.016 | 0.035 | 0.018 | -0.035 | -0.004 | 0.138 |
| DMR139 | FAM110A | -0.002 | 0.020 | 0.021 | 0.803 | -0.016 | 0.013 | 0.856 |
| DMR140 | ZGPAT,LIME1 | -0.016 | 0.057 | 0.073 | 0.700 | -0.106 | 0.073 | 0.810 |
| DMR141 | NA | -0.016 | 0.060 | 0.076 | 0.518 | -0.071 | 0.038 | 0.712 |
| DMR142 | SEPT5,GP1BB,SEPT5-GP1BB | 0.018 | 0.045 | 0.027 | 0.309 | -0.018 | 0.053 | 0.586 |
| DMR143 | LOC388849 | -0.077 | 0.046 | 0.123 | 0.038 | -0.148 | -0.005 | 0.176 |
| DMR144 | NA | 0.032 | 0.051 | 0.019 | 0.024 | 0.005 | 0.059 | 0.138 |
| DMR145 | TTLL8 | -0.035 | 0.022 | 0.058 | 0.027 | -0.065 | -0.005 | 0.138 |
| DMR146 | CPT1B,CHKB-CPT1B,CHKB | -0.065 | 0.022 | 0.087 | 0.010 | -0.110 | -0.020 | 0.138 |

P values from two-sided T-test. All differences and mean values are beta values. FDR, false discovery rate p value.

|  | ALL | AML | Lymphoma | Solid |
| --- | --- | --- | --- | --- |
| ALL | - |  |  |  |
| AML | 0.71 | - |  |  |
| Lymphoma | 0.55 | 0.55 | - |  |
| Solid | 0.58 | 0.74 | 0.79 | - |

Supplementary Table 5 Correlations between DNA methylation at DMRs across the cancer types (study 1).

Supplementary Table 6 Genomic locations of CpGs in DMRs and DMPs and comparisons

| Location | EPIC | | All DMRs  (146 DMRs) | | Retained DMRs  (107 DMRs) | | Chi2 p value | | |
| --- | --- | --- | --- | --- | --- | --- | --- | --- | --- |
|  |  |  |  |  |  |  | EPIC vs all DMRs | EPIC vs retained DMRs | All DMRs vs retained DMRs |
|  | Freq | % | Freq | % | Freq | % |  |  |  |
| **TSS1500** | 126617 | 19.80 | 214 | 19.62 | 100 | 16.75 | 0.881 | 0.062 | 0.632 |
| **TSS200** | 80472 | 12.58 | 222 | 20.35 | 123 | 20.60 | **< 0.001** | **< 0.001** | 0.375 |
| **1^st^ Exon** | 47310 | 7.40 | 115 | 10.54 | 66 | 11.06 | **< 0.001** | **< 0.001** | 0.129 |
| **Body** | 360719 | 56.40 | 493 | 45.19 | 285 | 47.74 | **< 0.001** | **< 0.001** | 0.129 |
| **3'UTR** | 24456 | 3.82 | 47 | 4.31 | 23 | 3.85 | 0.405 | 0.9711 | 0.781 |
|  |  |  |  |  |  |  |  |  |  |
| **Total** | 639574 | 100 | 1091 | 100 | 597 | 100 |  |  |  |

Loci with more than 1 genomic region annotation could belong to >1 category. EPIC, All CpGs on the EPIC array mapping to the locations; All DMRs, all the CpGs within the 146 DMRs; Retained DMRs, all the CpGs within the 107 retained DMRs; UTR, untranslated region; TSS, transcriptional start site.

Supplementary Table 7 Locations of CpGs in the EPIC array, DMRs (all) and retained DMRs relative to islands and tests for differences in proportions

| **Relation to**  **Island** | EPIC array CpGs | All DMRs (146 DMRs) | Retained DMRs (107 DMRs) |  | EPIC array vs all DMRs | EPIC array vs retained DMRs | All DMRs vs retained DMRs |
| --- | --- | --- | --- | --- | --- | --- | --- |
| Island |  |  |  |  | **< 0.001** | **< 0.001** | 0.070 |
| No | 704418 (81.4%) | 620 (59.3%) | 315 (54.7%) |  |  |  |  |
| Yes | 161441 (18.6%) | 425 (40.7%) | 261 (45.3%) |  |  |  |  |
| NorthShelf |  |  |  |  | 0.115 | 0.777 | 0.433 |
| No | 833868 (96.3%) | 1016 (97.2%) | 556 (96.5%) |  |  |  |  |
| Yes | 31991 (3.7%) | 29 (2.8%) | 20 (3.5%) |  |  |  |  |
| NorthShore |  |  |  |  | **< 0.001** | **< 0.001** | 0.188 |
| No | 782495 (90.4%) | 876 (83.8%) | 497 (86.3%) |  |  |  |  |
| Yes | 83364 (9.6%) | 169 (16.2%) | 79 (13.7%) |  |  |  |  |
| OpenSea |  |  |  |  | **< 0.001** | **< 0.001** | 0.370 |
| No | 377678 (43.6%) | 809 (77.4%) | 457 (79.3%) |  |  |  |  |
| Yes | 488181 (56.4%) | 236 (22.6%) | 119 (20.7%) |  |  |  |  |
| SouthShelf |  |  |  |  | **0.007** | 0.276 | 0.360 |
| No | 836159 (96.6%) | 1025 (98.1%) | 561 (97.4%) |  |  |  |  |
| Yes | 29700 (3.4%) | 20 (1.9%) | 15 (2.6%) |  |  |  |  |
| SouthShore |  |  |  |  | **< 0.001** | **< 0.001** | 0.377 |
| No | 794677 (91.8%) | 879 (84.1%) | 494 (85.8%) |  |  |  |  |
| Yes | 71182 (8.2%) | 166 (15.9%) | 82 (14.2%) |  |  |  |  |

All EPIC CPGs (N=865859), all CpGs in DMRs (n=1045), all CpGs in retained DMRs (n= 576). All DMRs represent the CpGs in DMR that were differentially methylated in early vs late remission in childhood cancer patients (study 1).

Supplementary Table 8 Coherence of the direction of DNA methylation change and the significance in adult CCS (study 2) relative to the differential methylation in early vs late remission (childhood patients, study 1) for DMPs and DMRs

|  | Direction | Not significant | | Significant | | χ² p |
| --- | --- | --- | --- | --- | --- | --- |
|  |  | n | % | n | % |  |
| **DMPs** |  |  |  |  |  |  |
|  | Total | 515 |  | 697 |  | <0.001 |
|  | Different | 209 | 17.24 | 119 | 9.82 |  |
|  | Same | 306 | 25.25 | 578 | 47.69 |  |
| **DMR CpGs** |  |  |  |  |  |  |
|  | Total | 303 |  | 739 |  | <0.001 |
|  | Different | 87 | 8.35 | 51 | 4.89 |  |
|  | Same | 216 | 20.73 | 688 | 66.03 |  |

Total n=2,253. % represent percentage of the total. Significantly different refers to difference between cases and controls (determined from T-test FDR value<0.05). χ² p is testing the association between the direction and significance in the two-way table.

**Supplementary Table 9 Adjusted linear models of DMR (n=107) methylation associated with adult CCS cases (study 2) vs adult controls (CCs n=32; controls, n=284), treatment (just lymphoma survivors, n=29): IR vs chemo, and testicular cases vs lymphoma (n=32).**

|  |  | Cases vs control | | | | | |  | IR vs chemo (lymphoma survivors) | | | | | |  | Testicular vs lymphoma | | | | | |
| --- | --- | --- | --- | --- | --- | --- | --- | --- | --- | --- | --- | --- | --- | --- | --- | --- | --- | --- | --- | --- | --- |
| DMR | Nearest Gene | 0 | Lower CI | Upper CI | R2 | p | FDR |  | Estimate | Lower CI | Upper CI | R2 | p | FDR |  | Estimate | Lower CI | Upper CI | R2 | p | FDR |
| Average | - | 0.043 | 0.036 | 0.05 | 0.788 | <0.001 | <0.001 |  | -0.003 | -0.027 | 0.021 | 0.618 | 0.785 | 0.993 |  | -0.016 | -0.051 | 0.019 | 0.631 | 0.354 | 0.727 |
| DMR1 | ACAP3 | 0.019 | 0.006 | 0.032 | 0.469 | 0.004 | 0.005 |  | -0.022 | -0.072 | 0.029 | 0.323 | 0.377 | 0.993 |  | 0.047 | -0.024 | 0.119 | 0.366 | 0.181 | 0.638 |
| DMR2 | MIB2 | 0.075 | 0.05 | 0.1 | 0.331 | <0.001 | <0.001 |  | 0.029 | -0.042 | 0.099 | 0.287 | 0.408 | 0.993 |  | 0.022 | -0.093 | 0.136 | 0.29 | 0.698 | 0.915 |
| DMR3 | NA | 0.039 | 0.017 | 0.062 | 0.343 | 0.001 | 0.001 |  | -0.02 | -0.069 | 0.028 | 0.563 | 0.397 | 0.993 |  | -0.068 | -0.14 | 0.003 | 0.562 | 0.06 | 0.540 |
| DMR4 | FLJ42875,PRDM16 | 0.065 | 0.057 | 0.074 | 0.629 | <0.001 | <0.001 |  | -0.011 | -0.04 | 0.018 | 0.772 | 0.443 | 0.993 |  | -0.038 | -0.085 | 0.01 | 0.743 | 0.116 | 0.594 |
| DMR5 | PRDM16 | 0.057 | 0.047 | 0.067 | 0.609 | <0.001 | <0.001 |  | -0.004 | -0.042 | 0.035 | 0.567 | 0.849 | 0.993 |  | -0.049 | -0.105 | 0.008 | 0.583 | 0.09 | 0.572 |
| DMR6 | PRDM16 | 0.061 | 0.046 | 0.076 | 0.456 | <0.001 | <0.001 |  | 0.004 | -0.039 | 0.047 | 0.485 | 0.848 | 0.993 |  | -0.054 | -0.123 | 0.015 | 0.469 | 0.119 | 0.594 |
| DMR7 | PRDM16 | 0.044 | 0.032 | 0.055 | 0.631 | <0.001 | <0.001 |  | -0.011 | -0.046 | 0.024 | 0.675 | 0.524 | 0.993 |  | -0.011 | -0.057 | 0.035 | 0.747 | 0.625 | 0.915 |
| DMR8 | PRDM16 | 0.066 | 0.056 | 0.076 | 0.704 | <0.001 | <0.001 |  | -0.013 | -0.057 | 0.031 | 0.586 | 0.546 | 0.993 |  | -0.021 | -0.083 | 0.042 | 0.608 | 0.495 | 0.876 |
| DMR10 | TP73 | 0.047 | 0.034 | 0.061 | 0.239 | <0.001 | <0.001 |  | -0.01 | -0.053 | 0.034 | 0.441 | 0.652 | 0.993 |  | -0.043 | -0.104 | 0.018 | 0.488 | 0.161 | 0.638 |
| DMR12 | LYPLA2 | 0.047 | 0.038 | 0.055 | 0.575 | <0.001 | <0.001 |  | 0.001 | -0.026 | 0.028 | 0.565 | 0.944 | 0.993 |  | -0.024 | -0.062 | 0.015 | 0.58 | 0.213 | 0.645 |
| DMR13 | MAP3K6 | 0.043 | 0.025 | 0.061 | 0.329 | <0.001 | <0.001 |  | -0.026 | -0.076 | 0.024 | 0.394 | 0.29 | 0.993 |  | -0.045 | -0.122 | 0.033 | 0.359 | 0.243 | 0.645 |
| DMR14 | TFAP2E | 0.055 | 0.042 | 0.069 | 0.594 | <0.001 | <0.001 |  | 0.01 | -0.035 | 0.055 | 0.534 | 0.65 | 0.993 |  | -0.019 | -0.092 | 0.054 | 0.49 | 0.594 | 0.915 |
| DMR15 | DNALI1 | 0.033 | 0.022 | 0.045 | 0.481 | <0.001 | <0.001 |  | -0.008 | -0.057 | 0.041 | 0.328 | 0.734 | 0.993 |  | -0.034 | -0.101 | 0.034 | 0.43 | 0.315 | 0.694 |
| DMR17 | TNR | 0.038 | 0.017 | 0.059 | 0.249 | 0.001 | 0.001 |  | -0.027 | -0.083 | 0.029 | 0.389 | 0.328 | 0.993 |  | 0.008 | -0.077 | 0.093 | 0.365 | 0.845 | 0.962 |
| DMR18 | NAV1 | 0.059 | 0.03 | 0.089 | 0.139 | <0.001 | <0.001 |  | 0.035 | -0.063 | 0.133 | 0.241 | 0.464 | 0.993 |  | -0.086 | -0.228 | 0.057 | 0.359 | 0.226 | 0.645 |
| DMR20 | UCN | 0.017 | 0.011 | 0.024 | 0.841 | <0.001 | <0.001 |  | 0.004 | -0.011 | 0.018 | 0.454 | 0.611 | 0.993 |  | 0.009 | -0.014 | 0.032 | 0.431 | 0.42 | 0.796 |
| DMR21 | KRTCAP3,NRBP1 | 0.061 | 0.021 | 0.101 | 0.194 | 0.003 | 0.003 |  | -0.056 | -0.15 | 0.038 | 0.473 | 0.23 | 0.993 |  | -0.113 | -0.252 | 0.025 | 0.479 | 0.103 | 0.594 |
| DMR22 | MEIS1 | 0.035 | 0.021 | 0.049 | 0.49 | <0.001 | <0.001 |  | 0.021 | -0.02 | 0.062 | 0.395 | 0.302 | 0.993 |  | -0.041 | -0.103 | 0.021 | 0.397 | 0.183 | 0.638 |
| DMR23 | IL1RL2 | 0.026 | 0.005 | 0.046 | 0.277 | 0.015 | 0.016 |  | 0.056 | 0.016 | 0.096 | 0.71 | 0.008 | 0.864 |  | 0.061 | -0.007 | 0.13 | 0.606 | 0.077 | 0.572 |
| DMR24 | NA | 0.031 | -0.006 | 0.068 | 0.15 | 0.104 | 0.105 |  | -0.013 | -0.085 | 0.059 | 0.304 | 0.701 | 0.993 |  | -0.118 | -0.22 | -0.016 | 0.443 | 0.026 | 0.456 |
| DMR25 | TNS1 | 0.049 | 0.035 | 0.063 | 0.315 | <0.001 | <0.001 |  | -0.025 | -0.076 | 0.026 | 0.433 | 0.32 | 0.993 |  | 0.001 | -0.069 | 0.071 | 0.496 | 0.974 | 0.992 |
| DMR26 | NA | 0.041 | 0.015 | 0.068 | 0.24 | 0.002 | 0.002 |  | -0.031 | -0.11 | 0.048 | 0.273 | 0.422 | 0.993 |  | -0.062 | -0.182 | 0.058 | 0.244 | 0.295 | 0.687 |
| DMR27 | WNT6 | 0.075 | 0.053 | 0.096 | 0.237 | <0.001 | <0.001 |  | 0.022 | -0.056 | 0.1 | 0.571 | 0.557 | 0.993 |  | 0.029 | -0.085 | 0.144 | 0.57 | 0.599 | 0.915 |
| DMR29 | DNAH1 | 0.043 | 0.025 | 0.061 | 0.424 | <0.001 | <0.001 |  | -0.003 | -0.064 | 0.059 | 0.413 | 0.928 | 0.993 |  | -0.028 | -0.119 | 0.064 | 0.398 | 0.538 | 0.908 |
| DMR34 | PRDM8 | 0.091 | 0.065 | 0.117 | 0.245 | <0.001 | <0.001 |  | -0.02 | -0.103 | 0.064 | 0.573 | 0.63 | 0.993 |  | -0.186 | -0.309 | -0.063 | 0.594 | 0.005 | 0.456 |
| DMR35 | NA | 0.046 | 0.03 | 0.061 | 0.702 | <0.001 | <0.001 |  | -0.03 | -0.068 | 0.007 | 0.51 | 0.105 | 0.993 |  | -0.039 | -0.096 | 0.018 | 0.483 | 0.173 | 0.638 |
| DMR37 | CMYA5 | 0.03 | 0.006 | 0.055 | 0.351 | 0.016 | 0.017 |  | -0.01 | -0.079 | 0.06 | 0.179 | 0.77 | 0.993 |  | -0.024 | -0.126 | 0.078 | 0.217 | 0.625 | 0.915 |
| DMR38 | SLC25A2 | 0.042 | 0.019 | 0.064 | 0.342 | <0.001 | <0.001 |  | -0.019 | -0.067 | 0.029 | 0.512 | 0.416 | 0.993 |  | -0.08 | -0.156 | -0.005 | 0.548 | 0.038 | 0.456 |
| DMR40 | NEURL1B | 0.049 | 0.036 | 0.062 | 0.545 | <0.001 | <0.001 |  | -0.015 | -0.058 | 0.029 | 0.442 | 0.492 | 0.993 |  | 0.013 | -0.05 | 0.076 | 0.445 | 0.667 | 0.915 |
| DMR41 | PFN3 | 0.03 | 0.013 | 0.047 | 0.479 | 0.001 | 0.001 |  | -0.005 | -0.048 | 0.038 | 0.599 | 0.811 | 0.993 |  | -0.037 | -0.1 | 0.027 | 0.614 | 0.248 | 0.645 |
| DMR45 | NA | 0.047 | 0.029 | 0.066 | 0.437 | <0.001 | <0.001 |  | -0.002 | -0.05 | 0.046 | 0.666 | 0.934 | 0.993 |  | 0 | -0.071 | 0.071 | 0.675 | 0.994 | 0.994 |
| DMR46 | KIAA1949,NRM | 0.031 | 0.026 | 0.036 | 0.904 | <0.001 | <0.001 |  | -0.004 | -0.019 | 0.011 | 0.723 | 0.575 | 0.993 |  | 0.003 | -0.019 | 0.025 | 0.734 | 0.771 | 0.915 |
| DMR47 | DDR1 | 0.045 | 0.032 | 0.059 | 0.39 | <0.001 | <0.001 |  | 0.005 | -0.025 | 0.035 | 0.632 | 0.732 | 0.993 |  | -0.012 | -0.056 | 0.031 | 0.638 | 0.555 | 0.915 |
| DMR48 | DDR1 | 0.028 | 0.022 | 0.035 | 0.652 | <0.001 | <0.001 |  | 0.004 | -0.008 | 0.016 | 0.514 | 0.52 | 0.993 |  | 0 | -0.017 | 0.018 | 0.509 | 0.971 | 0.992 |
| DMR50 | VARS | 0.045 | 0.032 | 0.058 | 0.576 | <0.001 | <0.001 |  | -0.033 | -0.084 | 0.017 | 0.518 | 0.182 | 0.993 |  | -0.012 | -0.083 | 0.059 | 0.559 | 0.723 | 0.915 |
| DMR51 | HLA-DPB1 | 0.037 | 0 | 0.073 | 0.159 | 0.049 | 0.051 |  | -0.01 | -0.109 | 0.089 | 0.105 | 0.838 | 0.993 |  | -0.175 | -0.323 | -0.027 | 0.319 | 0.022 | 0.456 |
| DMR52 | HLA-DPB2 | 0.038 | 0.019 | 0.058 | 0.104 | <0.001 | <0.001 |  | 0.002 | -0.064 | 0.068 | 0.258 | 0.959 | 0.993 |  | 0.015 | -0.084 | 0.114 | 0.389 | 0.756 | 0.915 |
| DMR53 | COL11A2 | 0.027 | 0.009 | 0.044 | 0.524 | 0.003 | 0.003 |  | 0.002 | -0.063 | 0.067 | 0.318 | 0.949 | 0.993 |  | 0.051 | -0.043 | 0.145 | 0.363 | 0.273 | 0.655 |
| DMR55 | MAD1L1 | 0.046 | 0.036 | 0.056 | 0.915 | <0.001 | <0.001 |  | -0.012 | -0.026 | 0.002 | 0.5 | 0.092 | 0.993 |  | -0.017 | -0.045 | 0.012 | 0.359 | 0.236 | 0.645 |
| DMR56 | RADIL | 0.026 | 0.013 | 0.038 | 0.118 | <0.001 | <0.001 |  | -0.005 | -0.03 | 0.02 | 0.502 | 0.693 | 0.993 |  | -0.021 | -0.061 | 0.02 | 0.479 | 0.307 | 0.691 |
| DMR57 | HOXA4 | 0.058 | 0.031 | 0.085 | 0.229 | <0.001 | <0.001 |  | -0.022 | -0.117 | 0.073 | 0.248 | 0.631 | 0.993 |  | -0.055 | -0.194 | 0.084 | 0.262 | 0.418 | 0.796 |
| DMR58 | HOXA5 | 0.025 | 0.004 | 0.046 | 0.366 | 0.018 | 0.019 |  | -0.034 | -0.09 | 0.021 | 0.193 | 0.215 | 0.993 |  | 0.022 | -0.059 | 0.104 | 0.233 | 0.574 | 0.915 |
| DMR59 | TNS3 | 0.055 | 0.045 | 0.066 | 0.445 | <0.001 | <0.001 |  | -0.023 | -0.077 | 0.03 | 0.531 | 0.372 | 0.993 |  | -0.067 | -0.143 | 0.01 | 0.557 | 0.083 | 0.572 |
| DMR60 | SAP25,LRCH4,ZASP | 0.037 | 0.03 | 0.043 | 0.892 | <0.001 | <0.001 |  | 0.01 | 0.001 | 0.019 | 0.552 | 0.036 | 0.993 |  | -0.002 | -0.017 | 0.014 | 0.471 | 0.803 | 0.933 |
| DMR61 | AGAP3 | 0.043 | 0.018 | 0.067 | 0.103 | 0.001 | 0.001 |  | 0.012 | -0.033 | 0.056 | 0.338 | 0.587 | 0.993 |  | 0.05 | -0.014 | 0.115 | 0.365 | 0.121 | 0.594 |
| DMR62 | PIWIL2 | 0.038 | 0.03 | 0.046 | 0.936 | <0.001 | <0.001 |  | -0.002 | -0.007 | 0.004 | 0.528 | 0.566 | 0.993 |  | 0.009 | 0.001 | 0.018 | 0.493 | 0.035 | 0.456 |
| DMR63 | LOC101929237,PEBP4 | 0.032 | 0.018 | 0.047 | 0.359 | <0.001 | <0.001 |  | 0.012 | -0.036 | 0.059 | 0.508 | 0.611 | 0.993 |  | -0.014 | -0.085 | 0.057 | 0.535 | 0.694 | 0.915 |
| DMR64 | TRAPPC9 | 0.031 | 0.011 | 0.052 | 0.081 | 0.003 | 0.003 |  | -0.025 | -0.074 | 0.025 | 0.34 | 0.307 | 0.993 |  | 0.054 | -0.024 | 0.132 | 0.401 | 0.162 | 0.638 |
| DMR66 | MAMDC2 | 0.041 | 0.034 | 0.049 | 0.612 | <0.001 | <0.001 |  | 0.007 | -0.019 | 0.033 | 0.376 | 0.579 | 0.993 |  | -0.005 | -0.043 | 0.032 | 0.4 | 0.768 | 0.915 |
| DMR68 | LHX6 | 0.036 | 0.006 | 0.066 | 0.051 | 0.021 | 0.022 |  | -0.034 | -0.14 | 0.073 | 0.255 | 0.515 | 0.993 |  | 0.014 | -0.138 | 0.167 | 0.285 | 0.846 | 0.962 |
| DMR70 | C10orf47 | 0.019 | -0.014 | 0.052 | 0.394 | 0.266 | 0.266 |  | 0.02 | -0.058 | 0.098 | 0.437 | 0.598 | 0.993 |  | 0.019 | -0.098 | 0.136 | 0.44 | 0.74 | 0.915 |
| DMR71 | ALOX5 | 0.033 | 0.02 | 0.047 | 0.472 | <0.001 | <0.001 |  | 0.026 | -0.01 | 0.061 | 0.397 | 0.154 | 0.993 |  | -0.008 | -0.07 | 0.054 | 0.379 | 0.803 | 0.933 |
| DMR72 | NA | 0.054 | 0.035 | 0.072 | 0.503 | <0.001 | <0.001 |  | -0.019 | -0.071 | 0.034 | 0.648 | 0.468 | 0.993 |  | 0.017 | -0.058 | 0.092 | 0.657 | 0.64 | 0.915 |
| DMR73 | ZNF503 | 0.04 | 0.029 | 0.052 | 0.583 | <0.001 | <0.001 |  | -0.001 | -0.033 | 0.031 | 0.593 | 0.946 | 0.993 |  | -0.041 | -0.086 | 0.005 | 0.639 | 0.078 | 0.572 |
| DMR75 | NA | 0.06 | 0.046 | 0.075 | 0.685 | <0.001 | <0.001 |  | -0.011 | -0.056 | 0.034 | 0.542 | 0.62 | 0.993 |  | -0.041 | -0.107 | 0.025 | 0.561 | 0.214 | 0.645 |
| DMR76 | NA | 0.043 | 0.026 | 0.06 | 0.604 | <0.001 | <0.001 |  | 0.037 | -0.009 | 0.082 | 0.686 | 0.106 | 0.993 |  | -0.053 | -0.12 | 0.014 | 0.675 | 0.115 | 0.594 |
| DMR77 | INPP5F | 0.051 | 0.035 | 0.066 | 0.556 | <0.001 | <0.001 |  | -0.01 | -0.06 | 0.041 | 0.418 | 0.691 | 0.993 |  | -0.025 | -0.098 | 0.049 | 0.439 | 0.494 | 0.876 |
| DMR79 | NA | 0.047 | 0.037 | 0.058 | 0.479 | <0.001 | <0.001 |  | 0.003 | -0.038 | 0.044 | 0.283 | 0.872 | 0.993 |  | -0.041 | -0.099 | 0.017 | 0.347 | 0.159 | 0.638 |
| DMR80 | NA | 0.047 | 0.028 | 0.066 | 0.195 | <0.001 | <0.001 |  | 0.026 | -0.026 | 0.078 | 0.407 | 0.31 | 0.993 |  | -0.058 | -0.141 | 0.025 | 0.405 | 0.162 | 0.638 |
| DMR81 | MRVI1 | 0.037 | 0.029 | 0.045 | 0.766 | <0.001 | <0.001 |  | -0.002 | -0.031 | 0.026 | 0.444 | 0.861 | 0.993 |  | 0.009 | -0.033 | 0.05 | 0.457 | 0.67 | 0.915 |
| DMR82 | PTPN5 | 0.034 | 0.027 | 0.042 | 0.797 | <0.001 | <0.001 |  | -0.018 | -0.042 | 0.006 | 0.348 | 0.125 | 0.993 |  | -0.004 | -0.043 | 0.036 | 0.346 | 0.855 | 0.962 |
| DMR83 | NRXN2 | 0.047 | 0.037 | 0.058 | 0.611 | <0.001 | <0.001 |  | -0.006 | -0.035 | 0.023 | 0.59 | 0.656 | 0.993 |  | 0.011 | -0.032 | 0.054 | 0.582 | 0.599 | 0.915 |
| DMR84 | LTBP3 | 0.055 | 0.034 | 0.076 | 0.262 | <0.001 | <0.001 |  | -0.008 | -0.063 | 0.047 | 0.366 | 0.764 | 0.993 |  | 0.025 | -0.055 | 0.105 | 0.388 | 0.518 | 0.888 |
| DMR87 | ATG16L2 | 0.06 | 0.029 | 0.091 | 0.279 | <0.001 | <0.001 |  | 0.007 | -0.074 | 0.089 | 0.507 | 0.857 | 0.993 |  | -0.033 | -0.151 | 0.085 | 0.521 | 0.571 | 0.915 |
| DMR89 | AGAP2 | 0.034 | 0.018 | 0.049 | 0.824 | <0.001 | <0.001 |  | 0.014 | -0.023 | 0.051 | 0.306 | 0.428 | 0.993 |  | 0.018 | -0.038 | 0.073 | 0.285 | 0.511 | 0.888 |
| DMR90 | DUSP6 | -0.061 | -0.079 | -0.044 | 0.367 | <0.001 | <0.001 |  | 0.016 | -0.069 | 0.101 | 0.482 | 0.697 | 0.993 |  | 0.046 | -0.079 | 0.17 | 0.493 | 0.457 | 0.851 |
| DMR91 | KDM2B | 0.079 | 0.057 | 0.102 | 0.314 | <0.001 | <0.001 |  | -0.001 | -0.056 | 0.053 | 0.588 | 0.96 | 0.993 |  | -0.093 | -0.168 | -0.019 | 0.655 | 0.016 | 0.456 |
| DMR92 | CRYL1 | 0.084 | 0.062 | 0.105 | 0.307 | <0.001 | <0.001 |  | 0.021 | -0.041 | 0.083 | 0.517 | 0.486 | 0.993 |  | 0.023 | -0.071 | 0.116 | 0.492 | 0.621 | 0.915 |
| DMR93 | TEX26-AS1,TEX26, C13orf26 | 0.023 | -0.005 | 0.051 | 0.321 | 0.104 | 0.105 |  | 0.009 | -0.068 | 0.087 | 0.127 | 0.804 | 0.993 |  | 0.008 | -0.116 | 0.133 | 0.117 | 0.89 | 0.974 |
| DMR95 | SLC8A3 | 0.034 | 0.013 | 0.055 | 0.179 | 0.001 | 0.001 |  | -0.033 | -0.075 | 0.009 | 0.513 | 0.115 | 0.993 |  | 0.072 | 0.003 | 0.141 | 0.537 | 0.043 | 0.464 |
| DMR96 | FOXN3 | 0.018 | 0.008 | 0.028 | 0.781 | <0.001 | <0.001 |  | 0.008 | -0.01 | 0.026 | 0.662 | 0.344 | 0.993 |  | 0.015 | -0.011 | 0.041 | 0.656 | 0.258 | 0.648 |
| DMR100 | SPTBN5 | 0.046 | 0.035 | 0.056 | 0.319 | <0.001 | <0.001 |  | 0 | -0.024 | 0.025 | 0.635 | 0.99 | 0.993 |  | 0.001 | -0.036 | 0.037 | 0.624 | 0.971 | 0.992 |
| DMR101 | STRA6 | 0.016 | 0.008 | 0.024 | 0.534 | <0.001 | <0.001 |  | 0.003 | -0.017 | 0.024 | 0.467 | 0.752 | 0.993 |  | -0.002 | -0.032 | 0.029 | 0.469 | 0.917 | 0.974 |
| DMR102 | RGMA | 0.04 | 0.018 | 0.061 | 0.328 | <0.001 | <0.001 |  | -0.011 | -0.083 | 0.061 | 0.35 | 0.746 | 0.993 |  | -0.065 | -0.17 | 0.041 | 0.421 | 0.216 | 0.645 |
| DMR103 | C16orf91 | 0.037 | 0.025 | 0.048 | 0.231 | <0.001 | <0.001 |  | 0.002 | -0.025 | 0.029 | 0.68 | 0.859 | 0.993 |  | 0.019 | -0.022 | 0.061 | 0.647 | 0.345 | 0.727 |
| DMR104 | MIR1225,PKD1 | 0.027 | 0.005 | 0.049 | 0.659 | 0.015 | 0.016 |  | -0.01 | -0.071 | 0.051 | 0.178 | 0.737 | 0.993 |  | 0.046 | -0.044 | 0.136 | 0.165 | 0.299 | 0.687 |
| DMR106 | CLDN9 | 0.032 | 0.009 | 0.055 | 0.342 | 0.006 | 0.007 |  | -0.03 | -0.082 | 0.022 | 0.427 | 0.249 | 0.993 |  | 0.049 | -0.037 | 0.136 | 0.371 | 0.251 | 0.645 |
| DMR107 | VASN,CORO7,CORO7-PAM16 | 0.043 | 0.035 | 0.052 | 0.594 | <0.001 | <0.001 |  | 0 | -0.028 | 0.027 | 0.452 | 0.986 | 0.993 |  | 0.003 | -0.036 | 0.042 | 0.464 | 0.866 | 0.964 |
| DMR108 | ABAT | 0.038 | 0.028 | 0.048 | 0.438 | <0.001 | <0.001 |  | -0.001 | -0.035 | 0.033 | 0.557 | 0.949 | 0.993 |  | -0.029 | -0.078 | 0.021 | 0.576 | 0.24 | 0.645 |
| DMR109 | PRSS36 | 0.033 | 0.015 | 0.052 | 0.501 | <0.001 | <0.001 |  | -0.019 | -0.07 | 0.031 | 0.517 | 0.429 | 0.993 |  | 0.015 | -0.059 | 0.089 | 0.51 | 0.679 | 0.915 |
| DMR110 | ITGAM | 0.048 | 0.037 | 0.059 | 0.44 | <0.001 | <0.001 |  | 0.023 | -0.024 | 0.071 | 0.54 | 0.32 | 0.993 |  | -0.068 | -0.138 | 0.002 | 0.56 | 0.056 | 0.540 |
| DMR111 | ADGRG1,GPR56 | 0.054 | 0.041 | 0.068 | 0.593 | <0.001 | <0.001 |  | 0.012 | -0.025 | 0.05 | 0.674 | 0.496 | 0.993 |  | -0.042 | -0.097 | 0.013 | 0.683 | 0.129 | 0.606 |
| DMR112 | KIFC3 | 0.035 | 0.013 | 0.056 | 0.361 | 0.002 | 0.002 |  | 0.03 | -0.015 | 0.075 | 0.503 | 0.182 | 0.993 |  | -0.023 | -0.091 | 0.046 | 0.491 | 0.495 | 0.876 |
| DMR113 | ZCCHC14 | 0.034 | 0.017 | 0.052 | 0.671 | <0.001 | <0.001 |  | -0.016 | -0.075 | 0.044 | 0.364 | 0.587 | 0.993 |  | 0.038 | -0.051 | 0.128 | 0.386 | 0.384 | 0.754 |
| DMR114 | ZFPM1 | 0.061 | 0.045 | 0.076 | 0.708 | <0.001 | <0.001 |  | -0.01 | -0.068 | 0.049 | 0.672 | 0.735 | 0.993 |  | 0.003 | -0.08 | 0.086 | 0.683 | 0.938 | 0.984 |
| DMR117 | ABR | 0.052 | 0.027 | 0.076 | 0.611 | <0.001 | <0.001 |  | -0.045 | -0.102 | 0.011 | 0.623 | 0.109 | 0.993 |  | -0.091 | -0.176 | -0.007 | 0.603 | 0.036 | 0.456 |
| DMR118 | P2RX5,P2RX5-TAX1BP3 | 0.031 | 0.013 | 0.05 | 0.191 | 0.001 | 0.001 |  | -0.002 | -0.048 | 0.044 | 0.311 | 0.933 | 0.993 |  | 0.013 | -0.061 | 0.087 | 0.266 | 0.728 | 0.915 |
| DMR120 | USP43 | 0.015 | 0.001 | 0.028 | 0.719 | 0.029 | 0.030 |  | 0.01 | -0.021 | 0.042 | 0.642 | 0.511 | 0.993 |  | 0.008 | -0.042 | 0.059 | 0.61 | 0.739 | 0.915 |
| DMR121 | SEZ6 | 0.043 | 0.031 | 0.055 | 0.545 | <0.001 | <0.001 |  | -0.004 | -0.039 | 0.031 | 0.383 | 0.809 | 0.993 |  | 0.001 | -0.053 | 0.054 | 0.373 | 0.984 | 0.993 |
| DMR122 | ARHGAP23 | 0.058 | 0.044 | 0.073 | 0.289 | <0.001 | <0.001 |  | -0.01 | -0.07 | 0.05 | 0.269 | 0.728 | 0.993 |  | -0.104 | -0.188 | -0.021 | 0.406 | 0.017 | 0.456 |
| DMR123 | TCAP,STARD3 | 0.039 | 0.03 | 0.049 | 0.424 | <0.001 | <0.001 |  | -0.009 | -0.03 | 0.012 | 0.79 | 0.391 | 0.993 |  | 0.019 | -0.008 | 0.046 | 0.83 | 0.166 | 0.638 |
| DMR124 | FAM171A2 | 0.056 | 0.032 | 0.079 | 0.371 | <0.001 | <0.001 |  | -0.001 | -0.069 | 0.067 | 0.486 | 0.982 | 0.993 |  | 0.022 | -0.085 | 0.128 | 0.532 | 0.677 | 0.915 |
| DMR125 | ITGA2B | 0.071 | 0.055 | 0.087 | 0.53 | <0.001 | <0.001 |  | 0.024 | -0.021 | 0.07 | 0.622 | 0.271 | 0.993 |  | 0.045 | -0.027 | 0.117 | 0.575 | 0.204 | 0.645 |
| DMR126 | HOXB7 | 0.054 | 0.033 | 0.075 | 0.294 | <0.001 | <0.001 |  | 0.044 | -0.021 | 0.109 | 0.441 | 0.172 | 0.993 |  | -0.017 | -0.118 | 0.085 | 0.443 | 0.739 | 0.915 |
| DMR128 | WFIKKN2 | 0.024 | 0.015 | 0.034 | 0.475 | <0.001 | <0.001 |  | -0.006 | -0.035 | 0.022 | 0.475 | 0.657 | 0.993 |  | -0.002 | -0.044 | 0.039 | 0.508 | 0.907 | 0.974 |
| DMR129 | CACNG4 | 0.032 | 0.011 | 0.052 | 0.314 | 0.002 | 0.002 |  | 0.019 | -0.033 | 0.071 | 0.532 | 0.451 | 0.993 |  | 0.005 | -0.087 | 0.096 | 0.457 | 0.92 | 0.974 |
| DMR130 | NA | 0.035 | 0.02 | 0.049 | 0.657 | <0.001 | <0.001 |  | -0.018 | -0.054 | 0.018 | 0.52 | 0.307 | 0.993 |  | -0.03 | -0.081 | 0.022 | 0.57 | 0.243 | 0.645 |
| DMR132 | FAM38B | 0.076 | 0.052 | 0.101 | 0.282 | <0.001 | <0.001 |  | -0.002 | -0.07 | 0.065 | 0.493 | 0.944 | 0.993 |  | 0.046 | -0.054 | 0.146 | 0.495 | 0.352 | 0.727 |
| DMR134 | LINGO3 | 0.062 | 0.031 | 0.092 | 0.117 | <0.001 | <0.001 |  | -0.008 | -0.081 | 0.066 | 0.385 | 0.831 | 0.993 |  | 0.048 | -0.061 | 0.158 | 0.43 | 0.371 | 0.742 |
| DMR136 | CCER2 | 0.054 | 0.037 | 0.071 | 0.347 | <0.001 | <0.001 |  | -0.015 | -0.058 | 0.028 | 0.671 | 0.47 | 0.993 |  | -0.012 | -0.076 | 0.051 | 0.682 | 0.686 | 0.915 |
| DMR138 | MYBPC2 | 0.024 | 0.013 | 0.034 | 0.439 | <0.001 | <0.001 |  | -0.028 | -0.057 | 0 | 0.653 | 0.054 | 0.993 |  | -0.003 | -0.048 | 0.043 | 0.595 | 0.909 | 0.974 |
| DMR139 | FAM110A | 0.046 | 0.028 | 0.063 | 0.424 | <0.001 | <0.001 |  | 0.002 | -0.059 | 0.064 | 0.424 | 0.934 | 0.993 |  | -0.077 | -0.167 | 0.012 | 0.44 | 0.086 | 0.572 |
| DMR140 | ZGPAT,LIME1 | 0.066 | 0.055 | 0.077 | 0.753 | <0.001 | <0.001 |  | 0.002 | -0.03 | 0.034 | 0.752 | 0.896 | 0.993 |  | -0.022 | -0.072 | 0.027 | 0.729 | 0.357 | 0.727 |
| DMR142 | SEPT5,GP1BB | 0.044 | 0.031 | 0.057 | 0.256 | <0.001 | <0.001 |  | 0 | -0.03 | 0.03 | 0.617 | 0.993 | 0.993 |  | -0.007 | -0.05 | 0.037 | 0.626 | 0.756 | 0.915 |
| DMR143 | LOC388849 | 0.059 | 0.042 | 0.076 | 0.371 | <0.001 | <0.001 |  | 0.012 | -0.05 | 0.075 | 0.322 | 0.686 | 0.993 |  | -0.017 | -0.114 | 0.081 | 0.279 | 0.726 | 0.915 |
| DMR144 | NA | 0.035 | -0.001 | 0.071 | 0.381 | 0.058 | 0.060 |  | 0.002 | -0.096 | 0.099 | 0.43 | 0.973 | 0.993 |  | 0.077 | -0.062 | 0.215 | 0.467 | 0.264 | 0.648 |
| DMR146 | CPT1B,CHKB-CPT1B,CHKB | 0.053 | 0.029 | 0.077 | 0.464 | <0.001 | <0.001 |  | 0.022 | -0.037 | 0.081 | 0.411 | 0.442 | 0.993 |  | -0.125 | -0.221 | -0.028 | 0.586 | 0.014 | 0.456 |

Case-control and testicular vs non-testicular models are adjusted for age, sex, and cell types (CD4T, CD8T, NK, B cell, Neutrophils, Monocytes). Pre vs post 1970 models adjusted for cell types and sex. Cases had decreased methylation at 1 DMR relative to controls; patients treated pre-1970 demonstrated decreased DNA methylation at 63 DMRs compared to those treated post-1970; testicular had decreased methylation in 61 DMRs relative to non-testicular.

Supplementary Table 10 Differences in DNA methylation age markers and surrogate markers between adult CCS (study 2) and matched controls (n CCS=32; n controls =284)

| Variable | Estimate | lower_ci | upper_ci | p_value | FDR | R^2^ |
| --- | --- | --- | --- | --- | --- | --- |
| Age Acceleration (pan-tissue) | 2.267 | 0.717 | 3.816 | 0.004 | **0.007** | 0.096 |
| GrimAge Acceleration | -0.029 | -1.676 | 1.617 | 0.972 | 0.999 | 0.08 |
| PhenoAge Acceleration | -0.001 | -2.205 | 2.202 | 0.999 | 0.999 | 0 |
| SkinBlood Age Acceleration | 5.468 | 4.324 | 6.613 | <0.001 | **<0.001** | 0.224 |
| IEAA | -0.001 | -1.431 | 1.43 | 0.999 | 0.999 | 0 |
| EEAA | -0.015 | -1.723 | 1.692 | 0.986 | 0.999 | 0.022 |
| DNA methylation-ADM | 18.102 | 12.808 | 23.396 | <0.001 | **<0.001** | 0.51 |
| DNA methylation-B2M | 92547.03 | 57238.97 | 127855.1 | <0.001 | **<0.001** | 0.471 |
| DNA methylation-CystatinC | 15177.04 | 6956.644 | 23397.43 | <0.001 | **<0.001** | 0.518 |
| DNA methylation-GDF15 | -21.747 | -60.3 | 16.807 | 0.268 | 0.357 | 0.411 |
| DNA methylation-Leptin | 1700.918 | 903.528 | 2498.309 | <0.001 | **<0.001** | 0.69 |
| DNA methylation-PACKYRS | -3.569 | -8.646 | 1.507 | 0.167 | 0.243 | 0.13 |
| DNA methylation-PAI1 | 1690.81 | 931.856 | 2449.764 | <0.001 | **<0.001** | 0.233 |
| DNA methylation-TIMP1 | 1215.571 | 933.319 | 1497.824 | <0.001 | **<0.001** | 0.651 |
| DNA methylation-TL | -0.244 | -0.311 | -0.178 | <0.001 | **<0.001** | 0.431 |

Estimates are differences in adult CCS relative to controls. All models are adjusted for age and sex. Ci, confidence interval; IEAA, intrinsic epigenetic age acceleration; EEAA, extrinsic epigenetic age acceleration; ADM, adrenomedullin; B2M, beta-2 microglobulin; CystatinC , Cystatin C; GDF15, growth differentiation factor 15; PACKYRS, the amount of cigarettes smoked; PAI1, plasminogen activation inhibitor 1, TIMP1, tissue inhibitor metalloproteinase 1; TL, telomere length.

Supplementary Table 11 Descriptive statistics of health outcomes of adult CCS (n=32, study 2) at follow-up, stratified by mortality and second malignancies at follow-up

|  |  | **Alive** | **Deceased** |  |  | **Cancer-free** | **Second malignancy** |  |
| --- | --- | --- | --- | --- | --- | --- | --- | --- |
|  | Total (N=32) | 0 (N=25) | 1 (N=7) | p value |  | 0 (N=22) | 1 (N=10) | p value |
| **Sex** |  |  |  | 0.454 |  |  |  | 0.355 |
| Female | 22 (68.8%) | 18 (72.0%) | 4 (57.1%) |  |  | 14 (63.6%) | 8 (80.0%) |  |
| Male | 10 (31.2%) | 7 (28.0%) | 3 (42.9%) |  |  | 8 (36.4%) | 2 (20.0%) |  |
| **Year diagnosed** |  |  |  | 0.499 |  |  |  | 0.412 |
| Mean (SD) | 1973.19 (7.240) | 1972.720 (7.514) | 1974.86(6.388) |  |  | 1973.91 (7.483) | 1971.60 (6.769) |  |
| Range | 1958 - 1986 | 1958 - 1983 | 1969 - 1986 |  |  | 1959 - 1986 | 1958- 1980 |  |
| **Pre-1970** |  |  |  | 0.459 |  |  |  | 0.66 |
| No | 24 (75.0%) | 18 (72.0%) | 6 (85.7%) |  |  | 16 (72.7%) | 8 (80.0%) |  |
| Yes | 8 (25.0%) | 7 (28.0%) | 1 (14.3%) |  |  | 6 (27.3%) | 2 (20.0%) |  |
| **Age diagnosed** |  |  |  | 0.699 |  |  |  | 0.699 |
| Mean (SD) | 20.36 (3.772) | 20.22 (3.848) | 20.86 (3.730) |  |  | 20.18 (4.054) | 20.75 (3.225) |  |
| Range | 10.91 - 24.94 | 10.91 - 24.94 | 14.39 - 24.40 |  |  | 10.91 - 24.94 | 15.65 - 24.40 |  |
| **Second malignancy** |  |  |  | 0.094 |  |  |  |  |
| No | 22 (68.8%) | 19 (76.0%) | 3 (42.9%) |  |  | - | - |  |
| Yes | 10 (31.2%) | 6 (24.0%) | 4 (57.1%) |  |  | - | - |  |
| **Deceased (follow-up)** |  |  |  |  |  |  |  |  |
| No | - | - | - |  |  | 19 (86.4%) | 6 (60.0%) | 0.094 |
| Yes | - | - | - |  |  | 3 (13.6%) | 4 (40.0%) |  |

χ² test for categorical variables, two-sample t-test with equal variance for continuous variables.

Supplementary Table 12 Adjusted linear models of DMR methylation (DMRs n=107) and long-term health outcomes in adult CCS (n=32, study 2) including mortality and second cancers

|  |  | Mortality | | | | | |  | Second cancer | | | | | |
| --- | --- | --- | --- | --- | --- | --- | --- | --- | --- | --- | --- | --- | --- | --- |
| DMR | Gene symbol | Estimate | Lower CI | Upper CI | R2 | p | FDR |  | Estimate | Lower CI | Upper CI | R2 | p | FDR |
| Average | NA | 0.017 | -0.006 | 0.04 | 0.332 | 0.134 | 0.536 |  | -0.008 | -0.03 | 0.013 | 0.29 | 0.443 | 0.951 |
| DMR1 | ACAP3 | -0.013 | -0.055 | 0.028 | 0.086 | 0.519 | 0.824 |  | -0.018 | -0.055 | 0.02 | 0.101 | 0.345 | 0.951 |
| DMR2 | MIB2 | 0.048 | -0.013 | 0.109 | 0.142 | 0.116 | 0.536 |  | -0.005 | -0.063 | 0.054 | 0.062 | 0.869 | 0.958 |
| DMR3 | NA | 0.027 | -0.019 | 0.074 | 0.214 | 0.239 | 0.628 |  | -0.007 | -0.051 | 0.037 | 0.177 | 0.741 | 0.951 |
| DMR4 | FLJ42875,PRDM16 | 0.021 | -0.018 | 0.06 | 0.274 | 0.273 | 0.650 |  | -0.006 | -0.043 | 0.03 | 0.245 | 0.73 | 0.951 |
| DMR5 | PRDM16 | 0.002 | -0.034 | 0.039 | 0.257 | 0.892 | 0.963 |  | -0.01 | -0.043 | 0.024 | 0.266 | 0.557 | 0.951 |
| DMR6 | PRDM16 | 0.019 | -0.021 | 0.06 | 0.212 | 0.342 | 0.705 |  | -0.006 | -0.044 | 0.032 | 0.189 | 0.739 | 0.951 |
| DMR7 | PRDM16 | 0.018 | -0.014 | 0.049 | 0.499 | 0.256 | 0.628 |  | -0.02 | -0.048 | 0.008 | 0.512 | 0.157 | 0.951 |
| DMR8 | PRDM16 | 0.03 | -0.007 | 0.067 | 0.417 | 0.109 | 0.536 |  | -0.023 | -0.057 | 0.012 | 0.399 | 0.187 | 0.951 |
| DMR10 | TP73 | 0.023 | -0.014 | 0.06 | 0.2 | 0.216 | 0.614 |  | 0.002 | -0.034 | 0.037 | 0.155 | 0.927 | 0.985 |
| DMR12 | LYPLA2 | 0.021 | -0.005 | 0.047 | 0.194 | 0.109 | 0.536 |  | -0.003 | -0.028 | 0.021 | 0.117 | 0.786 | 0.951 |
| DMR13 | MAP3K6 | -0.006 | -0.052 | 0.041 | 0.024 | 0.801 | 0.951 |  | 0.023 | -0.018 | 0.065 | 0.066 | 0.259 | 0.951 |
| DMR14 | TFAP2E | 0.003 | -0.045 | 0.052 | 0.025 | 0.885 | 0.963 |  | -0.01 | -0.054 | 0.035 | 0.031 | 0.664 | 0.951 |
| DMR15 | DNALI1 | 0.01 | -0.029 | 0.048 | 0.22 | 0.611 | 0.880 |  | -0.006 | -0.041 | 0.029 | 0.216 | 0.731 | 0.951 |
| DMR17 | TNR | 0.02 | -0.025 | 0.064 | 0.261 | 0.376 | 0.738 |  | -0.01 | -0.051 | 0.031 | 0.246 | 0.628 | 0.951 |
| DMR18 | NAV1 | 0.013 | -0.067 | 0.093 | 0.152 | 0.741 | 0.939 |  | -0.04 | -0.111 | 0.032 | 0.187 | 0.264 | 0.951 |
| DMR20 | UCN | 0.011 | -0.003 | 0.024 | 0.178 | 0.113 | 0.536 |  | -0.003 | -0.016 | 0.009 | 0.109 | 0.586 | 0.951 |
| DMR21 | KRTCAP3,NRBP1 | 0.034 | -0.051 | 0.119 | 0.156 | 0.422 | 0.772 |  | 0.024 | -0.055 | 0.103 | 0.147 | 0.543 | 0.951 |
| DMR22 | MEIS1 | 0.002 | -0.036 | 0.04 | 0.032 | 0.933 | 0.967 |  | -0.019 | -0.053 | 0.015 | 0.076 | 0.257 | 0.951 |
| DMR23 | IL1RL2 | 0.011 | -0.034 | 0.057 | 0.262 | 0.611 | 0.880 |  | -0.047 | -0.085 | -0.009 | 0.394 | 0.017 | 0.756 |
| DMR24 | NA | 0.031 | -0.03 | 0.093 | 0.142 | 0.306 | 0.681 |  | -0.01 | -0.067 | 0.048 | 0.112 | 0.727 | 0.951 |
| DMR25 | TNS1 | 0.051 | 0.016 | 0.086 | 0.459 | 0.006 | 0.252 |  | -0.001 | -0.037 | 0.036 | 0.291 | 0.974 | 0.985 |
| DMR26 | NA | 0.012 | -0.053 | 0.077 | 0.047 | 0.712 | 0.938 |  | 0.04 | -0.018 | 0.098 | 0.106 | 0.168 | 0.951 |
| DMR27 | WNT6 | 0.025 | -0.051 | 0.1 | 0.209 | 0.506 | 0.824 |  | -0.036 | -0.104 | 0.033 | 0.228 | 0.295 | 0.951 |
| DMR29 | DNAH1 | 0.012 | -0.043 | 0.067 | 0.083 | 0.671 | 0.923 |  | 0.002 | -0.049 | 0.053 | 0.077 | 0.937 | 0.985 |
| DMR34 | PRDM8 | 0.085 | 0.001 | 0.169 | 0.199 | 0.047 | 0.461 |  | -0.03 | -0.112 | 0.052 | 0.094 | 0.456 | 0.951 |
| DMR35 | NA | 0.017 | -0.018 | 0.051 | 0.193 | 0.335 | 0.705 |  | 0.005 | -0.027 | 0.037 | 0.168 | 0.763 | 0.951 |
| DMR37 | CMYA5 | -0.002 | -0.056 | 0.052 | 0.07 | 0.951 | 0.967 |  | -0.032 | -0.08 | 0.016 | 0.128 | 0.186 | 0.951 |
| DMR38 | SLC25A2 | 0.018 | -0.034 | 0.071 | 0.086 | 0.474 | 0.824 |  | 0.019 | -0.029 | 0.066 | 0.09 | 0.428 | 0.951 |
| DMR40 | NEURL1B | 0.022 | -0.013 | 0.056 | 0.287 | 0.214 | 0.614 |  | -0.004 | -0.037 | 0.029 | 0.248 | 0.805 | 0.951 |
| DMR41 | PFN3 | 0.025 | -0.015 | 0.065 | 0.367 | 0.208 | 0.614 |  | 0.001 | -0.036 | 0.039 | 0.33 | 0.946 | 0.985 |
| DMR45 | NA | -0.006 | -0.056 | 0.044 | 0.32 | 0.8 | 0.951 |  | -0.036 | -0.079 | 0.008 | 0.381 | 0.103 | 0.951 |
| DMR46 | KIAA1949,NRM | 0.001 | -0.018 | 0.021 | 0.061 | 0.879 | 0.963 |  | -0.006 | -0.024 | 0.013 | 0.073 | 0.532 | 0.951 |
| DMR47 | DDR1 | 0.022 | -0.008 | 0.053 | 0.24 | 0.14 | 0.540 |  | 0.01 | -0.019 | 0.039 | 0.193 | 0.48 | 0.951 |
| DMR48 | DDR1 | 0.006 | -0.005 | 0.016 | 0.234 | 0.277 | 0.650 |  | -0.001 | -0.011 | 0.008 | 0.203 | 0.764 | 0.951 |
| DMR50 | VARS | 0.045 | 0.007 | 0.084 | 0.444 | 0.024 | 0.370 |  | 0.004 | -0.035 | 0.043 | 0.331 | 0.83 | 0.951 |
| DMR51 | HLA-DPB1 | 0.043 | -0.042 | 0.128 | 0.041 | 0.309 | 0.681 |  | 0.009 | -0.071 | 0.089 | 0.006 | 0.817 | 0.951 |
| DMR52 | HLA-DPB2 | 0.009 | -0.051 | 0.069 | 0.042 | 0.765 | 0.939 |  | 0.016 | -0.039 | 0.071 | 0.051 | 0.558 | 0.951 |
| DMR53 | COL11A2 | 0.067 | 0.016 | 0.117 | 0.214 | 0.012 | 0.259 |  | 0.022 | -0.03 | 0.073 | 0.034 | 0.396 | 0.951 |
| DMR55 | MAD1L1 | 0 | -0.015 | 0.016 | 0.183 | 0.954 | 0.967 |  | 0.006 | -0.008 | 0.02 | 0.204 | 0.393 | 0.951 |
| DMR56 | RADIL | 0.019 | -0.006 | 0.043 | 0.195 | 0.129 | 0.536 |  | 0 | -0.023 | 0.024 | 0.125 | 0.976 | 0.985 |
| DMR57 | HOXA4 | 0.063 | -0.006 | 0.133 | 0.208 | 0.074 | 0.536 |  | -0.01 | -0.078 | 0.057 | 0.114 | 0.755 | 0.951 |
| DMR58 | HOXA5 | -0.025 | -0.065 | 0.016 | 0.191 | 0.222 | 0.615 |  | -0.012 | -0.05 | 0.026 | 0.159 | 0.524 | 0.951 |
| DMR59 | TNS3 | 0.04 | -0.009 | 0.089 | 0.232 | 0.102 | 0.536 |  | -0.013 | -0.06 | 0.033 | 0.164 | 0.564 | 0.951 |
| DMR60 | SAP25,LRCH4,ZASP | -0.003 | -0.014 | 0.007 | 0.018 | 0.512 | 0.824 |  | -0.009 | -0.018 | 0 | 0.133 | 0.05 | 0.951 |
| DMR61 | AGAP3 | -0.009 | -0.047 | 0.03 | 0.033 | 0.654 | 0.917 |  | -0.015 | -0.05 | 0.02 | 0.052 | 0.388 | 0.951 |
| DMR62 | PIWIL2 | 0.003 | -0.002 | 0.008 | 0.243 | 0.294 | 0.676 |  | -0.002 | -0.006 | 0.003 | 0.225 | 0.506 | 0.951 |
| DMR63 | LOC101929237,PEBP4 | 0.037 | -0.011 | 0.085 | 0.085 | 0.129 | 0.536 |  | 0.018 | -0.028 | 0.064 | 0.028 | 0.421 | 0.951 |
| DMR64 | TRAPPC9 | -0.017 | -0.063 | 0.029 | 0.103 | 0.46 | 0.814 |  | -0.012 | -0.055 | 0.03 | 0.096 | 0.554 | 0.951 |
| DMR66 | MAMDC2 | 0.016 | -0.006 | 0.038 | 0.117 | 0.15 | 0.559 |  | 0.013 | -0.008 | 0.033 | 0.101 | 0.208 | 0.951 |
| DMR68 | LHX6 | 0.012 | -0.065 | 0.088 | 0.237 | 0.757 | 0.939 |  | -0.036 | -0.105 | 0.033 | 0.264 | 0.292 | 0.951 |
| DMR70 | C10orf47 | -0.017 | -0.089 | 0.055 | 0.102 | 0.636 | 0.904 |  | -0.074 | -0.134 | -0.014 | 0.261 | 0.018 | 0.756 |
| DMR71 | ALOX5 | 0.021 | -0.016 | 0.058 | 0.054 | 0.248 | 0.628 |  | -0.017 | -0.051 | 0.018 | 0.041 | 0.328 | 0.951 |
| DMR72 | NA | 0.014 | -0.032 | 0.06 | 0.446 | 0.547 | 0.841 |  | -0.021 | -0.063 | 0.021 | 0.459 | 0.321 | 0.951 |
| DMR73 | ZNF503 | 0.011 | -0.022 | 0.044 | 0.215 | 0.506 | 0.824 |  | -0.009 | -0.039 | 0.021 | 0.213 | 0.553 | 0.951 |
| DMR75 | NA | 0.035 | -0.01 | 0.081 | 0.119 | 0.122 | 0.536 |  | 0.004 | -0.039 | 0.048 | 0.041 | 0.837 | 0.951 |
| DMR76 | NA | -0.041 | -0.095 | 0.013 | 0.108 | 0.133 | 0.536 |  | -0.01 | -0.062 | 0.041 | 0.037 | 0.693 | 0.951 |
| DMR77 | INPP5F | 0.051 | 0.013 | 0.089 | 0.359 | 0.01 | 0.259 |  | -0.012 | -0.051 | 0.027 | 0.197 | 0.537 | 0.951 |
| DMR79 | NA | 0.035 | 0.005 | 0.065 | 0.273 | 0.023 | 0.370 |  | -0.01 | -0.04 | 0.019 | 0.139 | 0.478 | 0.951 |
| DMR80 | NA | 0.043 | -0.006 | 0.091 | 0.136 | 0.083 | 0.536 |  | -0.005 | -0.052 | 0.042 | 0.038 | 0.819 | 0.951 |
| DMR81 | MRVI1 | 0.007 | -0.017 | 0.03 | 0.25 | 0.551 | 0.841 |  | -0.008 | -0.03 | 0.013 | 0.257 | 0.436 | 0.951 |
| DMR82 | PTPN5 | 0.001 | -0.02 | 0.022 | 0.228 | 0.889 | 0.963 |  | 0.004 | -0.016 | 0.023 | 0.232 | 0.699 | 0.951 |
| DMR83 | NRXN2 | 0.005 | -0.025 | 0.034 | 0.145 | 0.754 | 0.939 |  | -0.008 | -0.036 | 0.019 | 0.155 | 0.529 | 0.951 |
| DMR84 | LTBP3 | 0.003 | -0.042 | 0.049 | 0.151 | 0.877 | 0.963 |  | -0.005 | -0.047 | 0.036 | 0.153 | 0.79 | 0.951 |
| DMR87 | ATG16L2 | -0.003 | -0.084 | 0.078 | 0.047 | 0.946 | 0.967 |  | -0.032 | -0.105 | 0.041 | 0.074 | 0.375 | 0.951 |
| DMR89 | AGAP2 | 0 | -0.031 | 0.031 | 0.063 | 0.991 | 0.991 |  | -0.019 | -0.047 | 0.008 | 0.129 | 0.156 | 0.951 |
| DMR90 | DUSP6 | -0.144 | -0.201 | -0.087 | 0.547 | **<0.001** | **<0.001** |  | -0.035 | -0.107 | 0.037 | 0.148 | 0.325 | 0.951 |
| DMR91 | KDM2B | 0.022 | -0.031 | 0.075 | 0.258 | 0.412 | 0.767 |  | -0.005 | -0.054 | 0.044 | 0.24 | 0.845 | 0.951 |
| DMR92 | CRYL1 | -0.011 | -0.064 | 0.043 | 0.286 | 0.688 | 0.923 |  | -0.028 | -0.076 | 0.02 | 0.317 | 0.241 | 0.951 |
| DMR93 | TEX26-AS1,TEX26,C13orf26 | -0.006 | -0.069 | 0.058 | 0.016 | 0.856 | 0.963 |  | -0.032 | -0.089 | 0.025 | 0.058 | 0.264 | 0.951 |
| DMR95 | SLC8A3 | 0.012 | -0.033 | 0.057 | 0.162 | 0.586 | 0.879 |  | -0.012 | -0.054 | 0.029 | 0.164 | 0.55 | 0.951 |
| DMR96 | FOXN3 | -0.001 | -0.02 | 0.018 | 0.239 | 0.892 | 0.963 |  | -0.017 | -0.033 | -0.001 | 0.347 | 0.04 | 0.951 |
| DMR100 | SPTBN5 | 0.012 | -0.014 | 0.038 | 0.206 | 0.346 | 0.705 |  | -0.006 | -0.03 | 0.018 | 0.188 | 0.609 | 0.951 |
| DMR101 | STRA6 | 0.013 | -0.006 | 0.033 | 0.089 | 0.178 | 0.614 |  | 0.003 | -0.015 | 0.022 | 0.032 | 0.707 | 0.951 |
| DMR102 | RGMA | 0.003 | -0.056 | 0.061 | 0.248 | 0.925 | 0.967 |  | -0.058 | -0.107 | -0.009 | 0.38 | 0.021 | 0.756 |
| DMR103 | C16orf91 | -0.01 | -0.041 | 0.021 | 0.163 | 0.502 | 0.824 |  | -0.01 | -0.038 | 0.019 | 0.164 | 0.486 | 0.951 |
| DMR104 | MIR1225,PKD1 | 0.029 | -0.016 | 0.075 | 0.091 | 0.195 | 0.614 |  | -0.006 | -0.049 | 0.037 | 0.036 | 0.78 | 0.951 |
| DMR106 | CLDN9 | 0.01 | -0.04 | 0.059 | 0.116 | 0.692 | 0.923 |  | 0.012 | -0.034 | 0.057 | 0.119 | 0.598 | 0.951 |
| DMR107 | VASN,CORO7,CORO7-PAM16 | 0.006 | -0.018 | 0.031 | 0.111 | 0.597 | 0.880 |  | -0.008 | -0.03 | 0.015 | 0.118 | 0.48 | 0.951 |
| DMR108 | ABAT | 0.013 | -0.022 | 0.047 | 0.121 | 0.459 | 0.814 |  | -0.006 | -0.038 | 0.026 | 0.108 | 0.699 | 0.951 |
| DMR109 | PRSS36 | -0.003 | -0.044 | 0.037 | 0.382 | 0.863 | 0.963 |  | -0.001 | -0.038 | 0.036 | 0.382 | 0.949 | 0.985 |
| DMR110 | ITGAM | 0.027 | -0.016 | 0.07 | 0.284 | 0.207 | 0.614 |  | -0.027 | -0.067 | 0.012 | 0.293 | 0.166 | 0.951 |
| DMR111 | ADGRG1,GPR56 | 0.045 | 0.004 | 0.086 | 0.241 | 0.032 | 0.420 |  | -0.012 | -0.053 | 0.029 | 0.115 | 0.558 | 0.951 |
| DMR112 | KIFC3 | 0.014 | -0.033 | 0.06 | 0.015 | 0.553 | 0.841 |  | 0.002 | -0.041 | 0.044 | 0.003 | 0.934 | 0.985 |
| DMR113 | ZCCHC14 | -0.007 | -0.058 | 0.044 | 0.167 | 0.774 | 0.939 |  | -0.014 | -0.06 | 0.032 | 0.175 | 0.538 | 0.951 |
| DMR114 | ZFPM1 | 0.011 | -0.051 | 0.073 | 0.245 | 0.726 | 0.939 |  | -0.025 | -0.081 | 0.032 | 0.263 | 0.378 | 0.951 |
| DMR117 | ABR | 0.027 | -0.029 | 0.084 | 0.243 | 0.329 | 0.705 |  | 0.005 | -0.048 | 0.057 | 0.217 | 0.859 | 0.956 |
| DMR118 | P2RX5,P2RX5-TAX1BP3 | -0.002 | -0.042 | 0.037 | 0.102 | 0.901 | 0.963 |  | 0.004 | -0.032 | 0.04 | 0.103 | 0.824 | 0.951 |
| DMR120 | USP43 | 0.004 | -0.026 | 0.034 | 0.409 | 0.767 | 0.939 |  | -0.009 | -0.037 | 0.018 | 0.417 | 0.498 | 0.951 |
| DMR121 | SEZ6 | 0.024 | -0.005 | 0.053 | 0.197 | 0.102 | 0.536 |  | 0.018 | -0.009 | 0.045 | 0.169 | 0.188 | 0.951 |
| DMR122 | ARHGAP23 | 0.05 | 0.003 | 0.098 | 0.193 | 0.039 | 0.421 |  | 0.005 | -0.042 | 0.052 | 0.06 | 0.813 | 0.951 |
| DMR123 | TCAP,STARD3 | -0.001 | -0.023 | 0.022 | 0.517 | 0.958 | 0.967 |  | -0.014 | -0.034 | 0.005 | 0.552 | 0.149 | 0.951 |
| DMR124 | FAM171A2 | 0.024 | -0.048 | 0.097 | 0.073 | 0.502 | 0.824 |  | 0.007 | -0.06 | 0.074 | 0.059 | 0.839 | 0.951 |
| DMR125 | ITGA2B | 0.019 | -0.027 | 0.064 | 0.276 | 0.412 | 0.767 |  | -0.015 | -0.057 | 0.027 | 0.273 | 0.461 | 0.951 |
| DMR126 | HOXB7 | 0.05 | -0.011 | 0.111 | 0.148 | 0.107 | 0.536 |  | 0.006 | -0.053 | 0.065 | 0.065 | 0.839 | 0.951 |
| DMR128 | WFIKKN2 | 0.013 | -0.009 | 0.036 | 0.384 | 0.242 | 0.628 |  | -0.009 | -0.029 | 0.012 | 0.368 | 0.409 | 0.951 |
| DMR129 | CACNG4 | 0.023 | -0.032 | 0.077 | 0.193 | 0.403 | 0.767 |  | -0.01 | -0.06 | 0.041 | 0.177 | 0.692 | 0.951 |
| DMR130 | NA | 0.031 | 0.002 | 0.059 | 0.434 | 0.035 | 0.420 |  | 0 | -0.029 | 0.028 | 0.335 | 0.988 | 0.988 |
| DMR132 | FAM38B | 0.044 | -0.013 | 0.101 | 0.311 | 0.126 | 0.536 |  | -0.021 | -0.075 | 0.033 | 0.267 | 0.433 | 0.951 |
| DMR134 | LINGO3 | -0.006 | -0.07 | 0.059 | 0.157 | 0.853 | 0.963 |  | -0.001 | -0.061 | 0.058 | 0.156 | 0.96 | 0.985 |
| DMR136 | CCER2 | 0.035 | -0.011 | 0.081 | 0.279 | 0.134 | 0.536 |  | -0.011 | -0.054 | 0.033 | 0.225 | 0.628 | 0.951 |
| DMR138 | MYBPC2 | 0.018 | -0.01 | 0.046 | 0.355 | 0.198 | 0.614 |  | 0.006 | -0.02 | 0.032 | 0.32 | 0.646 | 0.951 |
| DMR139 | FAM110A | 0.011 | -0.044 | 0.066 | 0.098 | 0.685 | 0.923 |  | -0.035 | -0.084 | 0.014 | 0.158 | 0.151 | 0.951 |
| DMR140 | ZGPAT,LIME1 | 0.054 | 0.016 | 0.093 | 0.312 | 0.007 | 0.252 |  | 0.001 | -0.039 | 0.041 | 0.105 | 0.975 | 0.985 |
| DMR142 | SEPT5,GP1BB,SEPT5-GP1BB | 0.013 | -0.016 | 0.041 | 0.326 | 0.365 | 0.730 |  | -0.006 | -0.032 | 0.02 | 0.311 | 0.657 | 0.951 |
| DMR143 | LOC388849 | 0.034 | -0.019 | 0.087 | 0.103 | 0.198 | 0.614 |  | -0.021 | -0.07 | 0.029 | 0.071 | 0.401 | 0.951 |
| DMR144 | NA | -0.05 | -0.12 | 0.021 | 0.408 | 0.163 | 0.587 |  | -0.031 | -0.098 | 0.035 | 0.385 | 0.342 | 0.951 |
| DMR146 | CPT1B,CHKB-CPT1B,CHKB | 0.035 | -0.026 | 0.096 | 0.285 | 0.254 | 0.628 |  | -0.022 | -0.079 | 0.035 | 0.267 | 0.431 | 0.951 |

All models are adjusted for age and sex. Associations for mortality (vs alive) and for second cancer (vs no second cancer) with methylation at retained DMRs (DMR=107). Ci, confidence interval; FDR, false discovery rate p value.

Supplementary Table 13 Descriptive statistics of DNA surrogate markers in adult CCS (n=32, study 2) at follow-up, stratified by adult mortality

|  | Alive (N=25) | Deceased (N=7) | Total (N=32) | p value |
| --- | --- | --- | --- | --- |
| **Age** |  |  |  | 0.679 |
| Mean (SD) | 42.95 (7.39) | 41.64 (6.98) | 42.66 (7.22) |  |
| Range | 23.8 - 59.0 | 28.1 - 49.8 | 23.80 - 59.0 |  |
| **Age Acceleration (pan-tissue)** |  |  |  | 0.738 |
| Mean (SD) | 2.69 (5.29) | 3.41 (3.72) | 2.84 (4.94) |  |
| Range | -7.08 - 13.70 | -1.95 - 8.17 | -7.08 - 13.70 |  |
| **GrimAge Acceleration** |  |  |  | **< 0.001** |
| Mean (SD) | -1.92 (3.53) | 6.84 (3.75) | 0.00 (5.09) |  |
| Range | -8.23 - 6.40 | 2.40 - 11.50 | -8.27 - 11.50 |  |
| **PhenoAge Acceleration** |  |  |  | 0.091 |
| Mean (SD) | -1.02 (6.62) | 3.64 (4.30) | -0.00 (6.43) |  |
| Range | -12.42 - 16.99 | -2.00 - 8.84 | -12.42 - 16.99 |  |
| **SkinBlood Age Acceleration** |  |  |  | 0.622 |
| Mean (SD) | 3.300(3.90) | 4.06 (1.90) | 3.47 (3.55) |  |
| Range | -2.86 - 14.37 | 1.992 - 6.550 | -2.86 - 14.37 |  |
| **IEAA** |  |  |  | 0.539 |
| Mean (SD) | -0.19 (3.28) | 0.67 (3.00) | -0.00 (3.20) |  |
| Range | -6.75 - 6.73 | -2.71 - 4.61 | -6.75 - 6.73 |  |
| **EEAA** |  |  |  | 0.343 |
| Mean (SD) | -0.44 (5.32) | 1.59 (2.90) | 0.00 (4.92) |  |
| Range | -10.05 - 12.92 | -2.03 - 5.78 | -10.05 - 12.92 |  |
| **DNA methylation-ADM** |  |  |  | 0.315 |
| Mean (SD) | 334.62 (20.73) | 343.55 (19.12) | 336.58 (20.43) |  |
| Range | 293.83 - 372.29 | 309.07 - 366.78 | 293.82 - 372.29 |  |
| **DNA methylation-B2M** |  |  |  | **0.027** |
| Mean (SD) | 1470284.24 (127888.02) | 1591913.01 (96750.66) | 1496890.54 (130704.62) |  |
| Range | 1144087.77 - 1745597.00 | 1457349.59 - 1727686.82 | 1144087.77 - 1745597.00 |  |
| **DNA methylation-CystatinC** |  |  |  | 0.18 |
| Mean (SD) | 547334.24 (28887.42) | 564077.09 (26933.54) | 550996.74 (28912.05) |  |
| Range | 472037.28 - 603377.66 | 527928.64 - 597423.03 | 472037.28 - 603377.66 |  |
| **DNA methylation-GDF15** |  |  |  | 0.274 |
| Mean (SD) | 741.32 (117.03) | 795.50 (99.62) | 753.17 (114.20) |  |
| Range | 400.30 - 964.66 | 606.14 - 939.79 | 400.30 - 964.66 |  |
| **DNA methylation-Leptin** |  |  |  | 0.93 |
| Mean (SD) | 10487.25 (3999.19) | 10642.32 (4352.05) | 10521.17 (4006.52) |  |
| Range | 2784.26 - 16799.00 | 4846.22 - 15520.18 | 2784.26 - 16799.00 |  |
| **DNA methylation-PACKYRS** |  |  |  | **< 0.001** |
| Mean (SD) | 4.47 (7.99) | 22.50 (13.20) | 8.42 (11.86) |  |
| Range | -7.79 - 33.16 | 6.35 - 38.87 | -7.79 - 38.87 |  |
| **DNA methylation-PAI1** |  |  |  | **0.006** |
| Mean (SD) | 16218.19 (2276.15) | 19123.71 (2499.379) | 16853.77 (2590.24) |  |
| Range | 12483.71 - 22077.45 | 15179.191 - 22318.12 | 12483.71 - 22318.12 |  |
| **DNA methylation-TIMP1** |  |  |  | 0.116 |
| Mean (SD) | 32490.55 (1205.01) | 33275.28 (780.25) | 32662.21 (1162.17) |  |
| Range | 29493.86 - 34702.33 | 32118.00 - 34316.48 | 29493.86 - 34702.33 |  |
| **DNA methylation-TL** |  |  |  | **0.005** |
| Mean (SD) | 6.97 (0.20) | 6.70 (0.21) | 6.91 (0.23) |  |
| Range | 6.45 - 7.37 | 6.42 - 7.00 | 6.42 - 7.37 |  |

P value from two-sample T-test. IEAA, intrinsic epigenetic age acceleration; EEAA, extrinsic epigenetic age acceleration; ADM, adrenomedullin; B2M, beta-2 microglobulin; CystatinC , Cystatin C; GDF15, growth differentiation factor 15; PACKYRS, the amount of cigarettes smoked; PAI1, plasminogen activation inhibitor 1, TIMP1, tissue inhibitor metalloproteinase 1; TL, telomere length.
